# Lipid metabolism-driven regulatory networks involved in cone senescence and seed maturation in pine (*Pinus massoniana*)

**DOI:** 10.1038/s42003-026-10267-z

**Published:** 2026-05-18

**Authors:** Yuanxiang Zhao, Sujie You, Xueying Li, Nana An, Haoyun Wang, Feng Wu

**Affiliations:** 1 Institute for Forest Resources and Environment of Guizhou, Guizhou Key Laboratory of Forest Cultivation in Plateau Mountain, Guizhou University, Guiyang, China; 2 College of Forestry, Guizhou University, Guiyang, China

**Keywords:** Plant physiology, Plant molecular biology

## Abstract

Pine cone ripening represents a senescence process during which seed maturity increases. However, the regulatory mechanisms underlying these divergent developmental trajectories are poorly defined. We have integrated physiological, transcriptomic, and lipidomic analyses of *Pinus massoniana* cones and seeds to construct co-expression networks to elucidate maturation mechanisms. We report seed germination capacity to increased markedly during cone color transition (green to brown) and plateau thereafter, while seed vigor peaked during cone cracking. Transcriptomic analysis indicates that lipid metabolic pathways were enriched during maturation, with α-linolenic, linoleic, and arachidonic acid metabolism enriched in cones, and glycerolipid, fatty acid metabolism, and fatty acid biosynthesis enriched in seeds. Lipidomic profiling reveals dynamic lipid reprogramming: cone senescence was associated with triglyceride accumulation and glycerophospholipid degradation, whereas seed maturation involved a shift in lipid classes, with triglycerides/diglycerides and glycerophospholipids showing distinct associations with germination capacity and vigor, respectively. A time-ordered co-expression network reveals key regulatory modules: senescence-associated factors (e.g., *ERF3*) are linked to dehydrogenases involved in membrane lipid homeostasis disruption correlated with cone aging. Conversely, core genes involved in lipid metabolism, hormone response factors, and key transcription factors (e.g., *bZIP1*) formed a central module that is closely associated with lipid fluxes and may contribute to the regulation of seed germination capacity and vigor. These findings inform optimal harvest timing for *P. massoniana* seeds and elucidate molecular mechanisms driving conifer reproductive development.

## Abstract (web summary)

An investigation of lipid metabolism-driven regulatory networks in pine sheds light on the distinct roles of triglycerides and glycerophospholipids in cone senescence and seed vigor acquisition.

## Introduction

Pine trees (*Pinus* spp.) represent a significant source of raw materials for various industrial applications, such as papermaking, construction, and resin extraction^1,2^. Their seeds represent a valuable biological resource with considerable development potential, being rich in lipids and a variety of unsaturated fatty acids such as linoleic acid (LA) and oleic acid^3–6^. Lipid accumulation within seeds is closely associated with germination vigor and seedling establishment^7–11^. For instance, In *Pinus koraiensis*^11^, transcriptomic analyses have revealed coordinated expression of fatty acid desaturases and acyl-transferases during late seed development, while in *Pinus tabuliformis*, lipid remodeling has been linked to the acquisition of desiccation tolerance^10^. However, existing research has mostly focused on seed developmental stages, with the metabolic changes that occur in cones during maturation often being neglected.

The maturation of conifer cones is a complex biological process, during which the scales exhibit characteristic senescence features in the later developmental stages, including a reduction in resin secretion and decreased adhesiveness^5,6^. These changes ultimately lead to scale cracking and seed release^12–14^. Prior to maturation, cones remain closed, effectively reducing seed loss from animal predation or premature germination^5,6,14^.

A rich array of lipid metabolites has been identified within the cones of various pine species(e.g., *Pinus mugo*, *Pinus nigra*, and *Pinus sylvestris*), and their composition changes dynamically across developmental stages^15,16^. Lipid metabolism plays an important role in regulating plant senescence, and involves processes such as lipid peroxidation, alterations in lipase activity, and imbalances in membrane phospholipids that can induce cellular structural degradation^17–19^. Accordingly, lipid metabolism may play an important role in the maturation and cracking of conifer cones. If so, the underlying mechanisms of this have yet to be systematically investigated. A comprehensive understanding of these processes could be leveraged to optimize seed development and harvesting timing to enhance seed quality and yield.

*Pinus massoniana* is an important species in afforestation projects in southern China. The normal maturation of its cones and seeds is directly related to forest regeneration and the production of superior timber varieties^20–22^. While correlations between lipid accumulation in *P. massoniana* seeds and their maturation process have been reported^23,24^, and patterns of change nutritional elements within cones and seeds during maturation have been elucidated, no comprehensive investigation of dynamic changes in the lipidome and transcriptome during cone- and seed-maturation processes has been undertaken^25,26^. Accordingly, the molecular mechanisms underlying lipid accumulation and their correlation with cone development remain unclear. Additionally, the gene networks that regulate coordination between cone dehiscence and seed maturation are not well characterized, which constrains the development of efficient harvesting strategies.

We integrate physiological, lipidomic, and transcriptomic approaches to systematically investigate the regulatory mechanisms underlying the maturation processes of *P. massoniana* cones and seeds. Our specific objectives are to: elucidate the temporal characteristics of lipid metabolism during cone senescence and seed vigor establishment; identify key genes and co-expression modules that regulate lipid metabolism in cones and seeds; and clarify the molecular relationships between lipid dynamics, cone cracking, and seed germination. We expect our findings to improve understanding of reproductive developmental mechanisms in pine trees and provide a theoretical foundation to enhance their seed quality and optimize harvest timing.

## Results

### Changes in cone morphology and seed viability during maturation

**Fig. 1:**
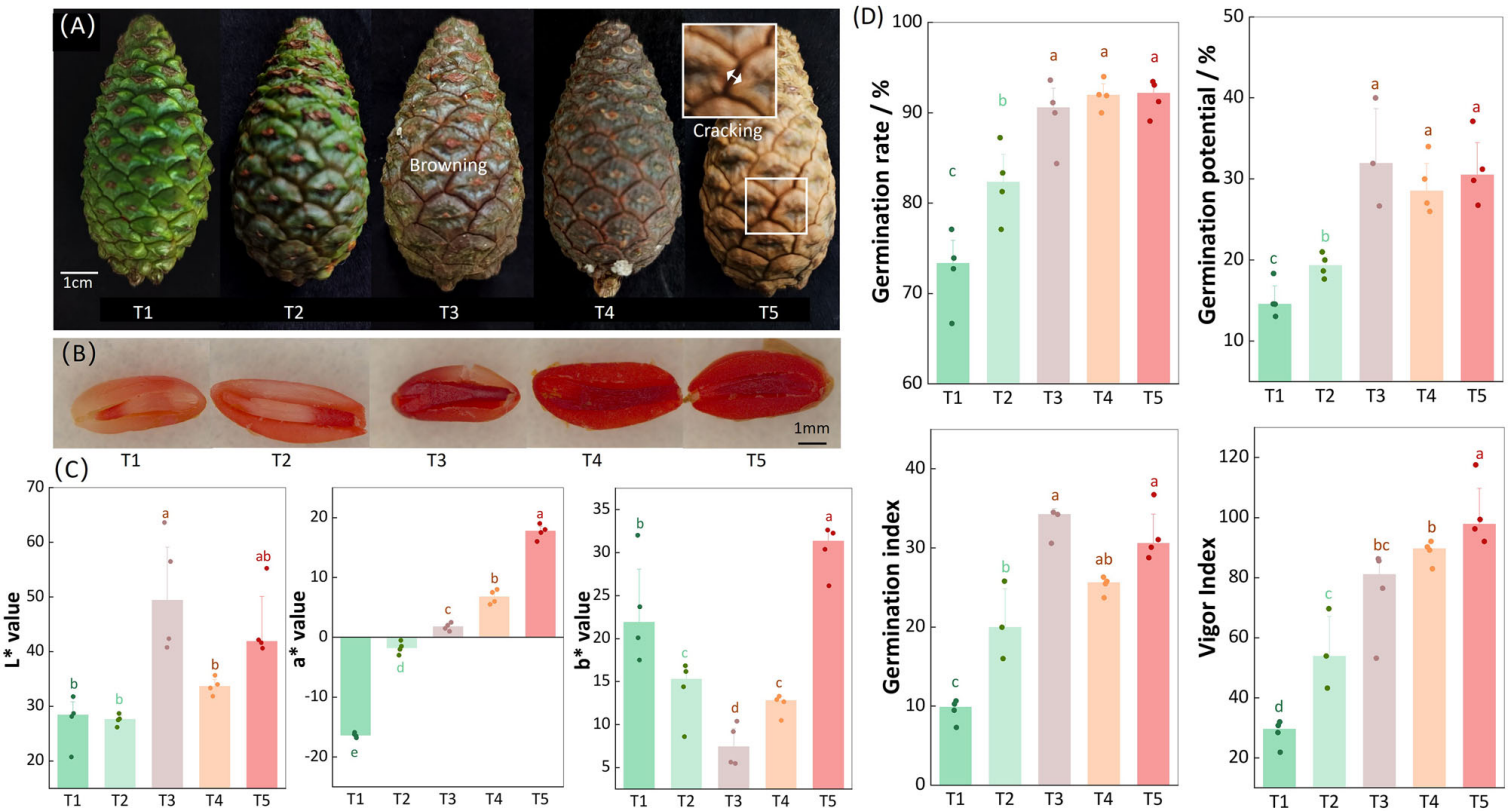
Morphological characteristics of *P. massoniana* cone and seed viability indicators during maturation. **A** The cone transition from green to gray-brown, and a widespread cracking event was observed at stage T5. **B** The 2,3,5-triphenyltetrazolium chloride (TTC) staining method indicates the maturity of seeds. **C** Temporal changes in chromaticity values (L*, a*, and b*). **D** Temporal dynamics of seed viability indicators, including germination rate (Gr), germination potential (Gp), germination index (Gi) and vigour index (Vi). Data are presented the mean ± SE (standard deviation) from four biological replicates (*n* = 4). Different letters denote statistically significant differences among stages (one-way ANOVA followed by Fisher’s least-significant-difference [LSD] test; *p* < 0.05).

Color is an important indicator of cone maturity^27^. Over time, the color of *P. massoniana* cones changed from green to gray-brown. Cracking of individual scales on cones occurred at the last experimental timepoint (T5); several other scales had begun to bend (Fig. 1A), indicating cones maturity. These observations are consistent with seed vigor as determined via 2,3,5-triphenyltetrazolium chloride (TTC) staining method (Fig. 1B). Additionally, the CIELAB color model of cones was scanned. From sampling points T1 to T5, no obvious change in L* values (brightness) occurred; the a* value (red and green attributes) gradually trended up, and the b* value (yellow and blue attributes) first decreased and then increased (Fig. 1C). This indicates that the increased a* value best characterized the cone color change from green to gray-brown. To improve the representativeness of the findings, concurrent temperature and precipitation data were collected from local monitoring stations (Supplementary Data 1). We replicated this observation in another clonal seed orchard of *P. massoniana* located in a state-owned forest farm in Duyun City, Guizhou Province, China, and recorded comparable patterns of change (Supplementary Fig 1). These results confirm that the color transition pattern is consistent across different environmental conditions.

Subsequently, we assessed the germination capacity of the seeds. The results, as illustrated in Fig. 1D, indicated that the seed germination rate (G_r_) gradually increased prior to the browning of the cones (T1-T3), rising from 77.08% to 91.11%, with a statistically significant difference (*p* < 0.05). Following the browning of the cones (T3-T5), the G_r_ began to stabilize. The seed germination potential (G_p_) and germination index (G_i_) exhibited similar trends, with G_p_ increasing from 15.12% to 32.86% prior to browning, and the G_i_ rising from 9.42 to 30.14, both stabilizing post-browning. This finding indicating that cone browning (corresponding to the T3 period) was a crucial marker of seed physiological maturity. Notably, although germination metrics ceased to increase at browning, the vigor index (V_i_) continued to rise afterward, peaking at 101.27 during cracking, suggesting that seed quality would further enhance following browning. Trends in seed germination parameters (G_r_, G_p_, G_i_) and vigor (V_i_) were supported by comparative data from another clonal seed orchard in Duyun (Supplementary Fig 1).

### Gene expression patterns in cones and seeds during maturation

**Fig. 2:**
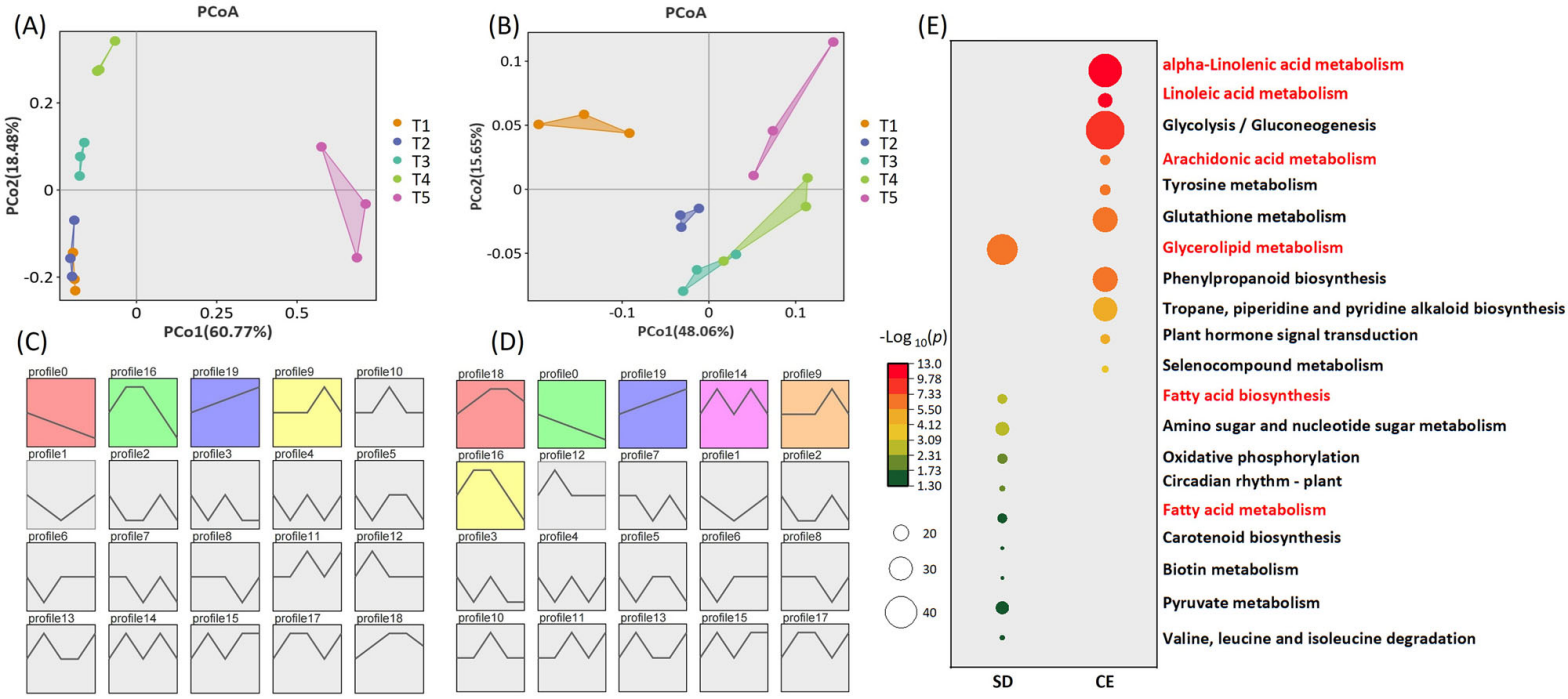
Gene expression patterns and KEGG functional enrichment in *P. massoniana* cones and seeds during maturation. Principal Co-ordinate Analysis (PCoA) of global gene-expression profiles for cones (**A**) and seeds (**B**) across developmental stages. Short Time-series Expression Miner (STEM) -style profile clustering of temporally expressed genes in cones (**C**) and seeds (**D**). Profiles are ordered by the significance (*P*-value) of the number of genes assigned versus the number expected by chance. Profiles shown on a coloured background are statistically significant (*P* < 0.05); profiles on a white background are not significant. Clusters sharing the same colour represent similar temporal expression trends. **E** Kyoto Encyclopedia of Genes and Genomes (KEGG) pathway enrichment for genes contained within the significant expression modules. Seed-derived modules (SD); Cone-derived modules (CE). Pathways are ranked by enrichment significance (adjusted *P*-value).

Thirty RNA-seq libraries from cones and seeds (averaging 48.8 million raw reads per library) were acquired. After filtering out low quality reads, the number of clean reads per library remained > 47.6 million (Supplementary Data 2). Approximately 70% of the reads could be unambiguously mapped to the *P. tabuliformis* reference coding sequence. Totals of 63,179 and 62,120 expressed genes were identified in cones and seeds, among which 13,544 and 6529 were differentially expressed genes (DEGs) during maturation (Supplementary Fig 2). Principal Co-ordinate Analysis (PCoA), based on DEG-related transcripts per kilobase million values (TPM) from the entire experiment revealed the global dynamics of gene expression. In seeds and cones, the first two PCo collectively accounted for 79.25% and 63.71% of all variance and showed gene expression groupings according to sampling time (Fig. 2A, B).

To identify gene clusters with similar expression dynamics, Short Time-series Expression Miner (STEM) software was used. DEGs from cones and seeds were partitioned into 20 distinct clusters (Supplementary Data 3 and 4), among which four for cones and six for seeds exhibited statistically significant expression trends (*p* < 0.05) (Fig. 2C, D). Functional enrichment analysis of Kyoto Encyclopedia of Genes and Genomes (KEGG) pathways revealed these dynamic gene modules to be strongly associated with lipid metabolism (Fig. 2E; Supplementary Data 5 and 6). Specifically, cone-specific clusters were linked to α-linolenic acid (ALA), LA, and arachidonic acid (AA) metabolisms. Seed-specific clusters were enriched in glycerolipid metabolism, fatty acids (FA) metabolism, and FA biosynthesis. These findings reveal lipid metabolic reprogramming to be a hallmark of cone and seed maturation, with tissue-specific pathway activation driving lipid accumulation.

### Lipid accumulation dynamics in cones and seeds during maturation

**Fig. 3:**
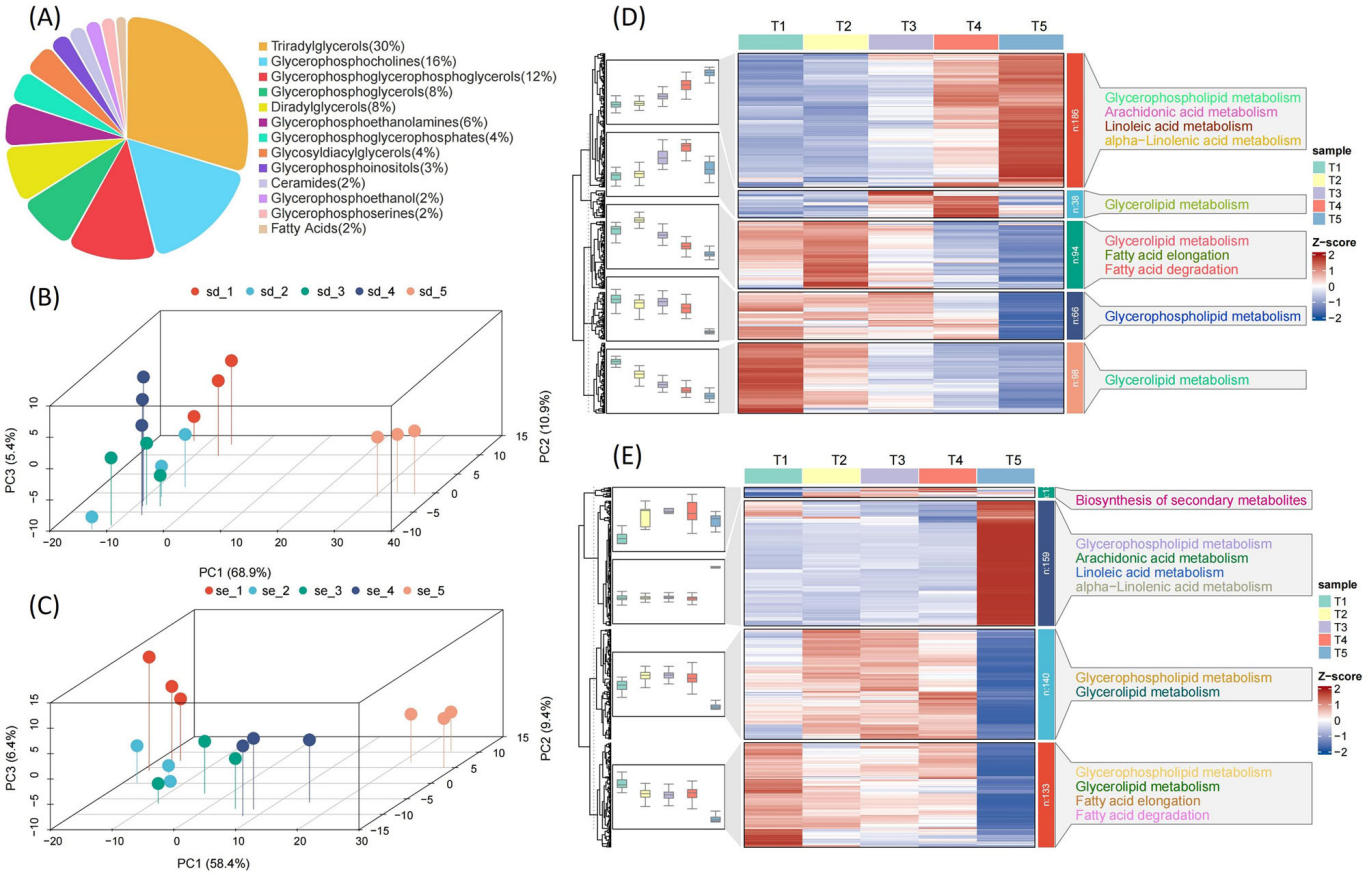
Lipid metabolic profiles of cones and seeds of *P. massoniana* during maturation. **A** Composition and relative proportions of detected lipid metabolites, grouped by lipid class. Principal component analysis (PCA) of lipidomic profiles for cones (**B**) and seeds (**C**) across developmental stages; the percentage of variance explained by each principal component is indicated on the axes. Heatmaps and hierarchical clustering of lipid metabolite abundances across five developmental stages in cones (**D**) and seeds (**E**). Values shown are Z-score normalized across rows (metabolites) so that colors reflect relative changes for each metabolite across stages; the colour scale runs from blue (low relative abundance) to red (high relative abundance).

Given the central role of lipid metabolism indicated by the transcriptome, we performed a comprehensive lipidomic analysis to directly quantify changes in the composition of lipids throughout maturation. Of 5164 lipid metabolites identified in cones and seeds during maturation, 3076 were detected in positive ion mode (and 2088 in negative ion mode). Chemical characteristics enabled 4417 (85.53% of all metabolites) annotated in the KEGG database (Supplementary Data 7) to be classified into 13 groups (Fig. 3A). The majority of lipid metabolites were triacylglycerols (TG, 30%), phosphatidylcholine (PC, 16%), and glycerophosphatidylglycerol (PG, 12%). Analysis of the metabolome using PCA revealed all cone and seed samples separated into five groups, indicating that accumulation of and changes in metabolites were dynamic during cone and seed maturation (Fig. 3B, C). To enhance group differentiation and identify differentially accumulated metabolites (DAMs), the OPLS–DA model was used to eliminate orthogonal variables that were irrelevant to the classification of metabolites. Of 367 DAMs identified in cones, 118 were upregulated, and of 651 DAMs identified in seeds, 316 were upregulated (Supplementary Fig. 3).

Of five DAM clusters in cones with significant expression trends (Fig. 3D) (identified by K-means clustering), two key expression patterns were prominent: 1) with a progressive upregulation from T1 to T5, primarily enriched in glycerophospholipid (GPL), AA, LA, and ALA metabolism; and 2) with a sustained downregulation in expression, with significant involvement in biological processes such as GPL metabolism, glycerolipid metabolism, FA degradation and elongation.

Seed DAMs formed four expression clusters (Fig. 3E), with two dominant patterns: 1) with minimal expression before maturation but a sharp increase at the final stage, with enriched pathways including GPL, AA, LA, and ALA metabolism; and 2), where metabolites were highly expressed in earlier stages but declined substantially upon maturation, primarily involved in GPL metabolism, glyceride metabolism, and FA degradation/elongation. Notably, metabolic enrichment pathways in cones overlapped significantly with those in seeds, although specificity in regulatory timing and metabolite composition occurred.

### Key DAMs associated with maturation in cones and seeds

**Fig. 4:**
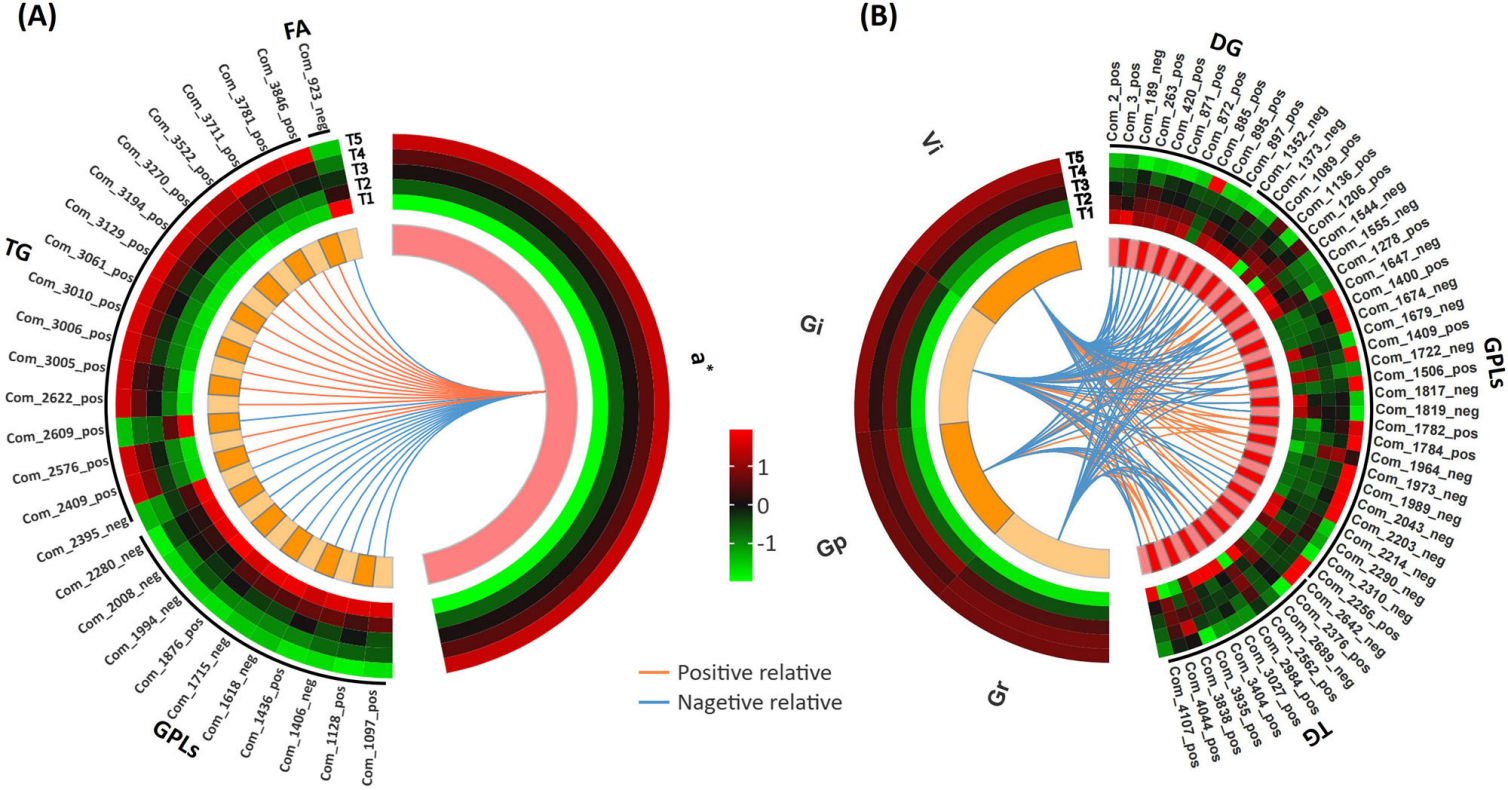
Correlations between differentially accumulated metabolites (DAMs) related to lipid metabolism and maturity indicators in *P. massoniana* cones and seeds. Heatmap showing the correlative relationships between DAMs linked to lipid metabolism and cone (**A**) and seed (**B**) maturity indicators. The heatmap depicts the after Z-score normalization across rows, with expression levels indicated by a color gradient ranging from green to red, representing low to high levels, respectively. The middle line represents the positive and negative correlations (|R | > 0.90). Red and blue represent positive and negative correlations, respectively.

Cone maturity (assessed by the a* value, Fig. 1B) correlated significantly (*R* > 0.9 and *p* < 0.001) with 27 DAMs (16 TGs, 10 GPLs, 1 FA). The a* value correlated positively with TG accumulation but negatively with GPLs and FA, indicating that lipid dynamics played important roles in cone maturation (Fig. 4A), potentially characterized by accumulation of TG and degradation of GPLs and FA.

Forty-nine DAMs were closely associated with seed vigor indices. Among them, G_r_, G_p_, and G_i_ were primarily related to TG and diglycerides (DGs) (positively and negatively), while V_i_ was predominantly associated with GPLs, including PC, phosphatidylethanolamine (PE), PG, and phosphoinositol (PI) (Fig. 4B). These results indicate that lipid classes are associated with seed quality traits, with TGs and DGs showing correlations with germination-related traits and GPLs being more closely associated with seed vigor.

### Time-ordered gene co-expression network (TO–GCN) related to lipid metabolism

**Fig. 5:**
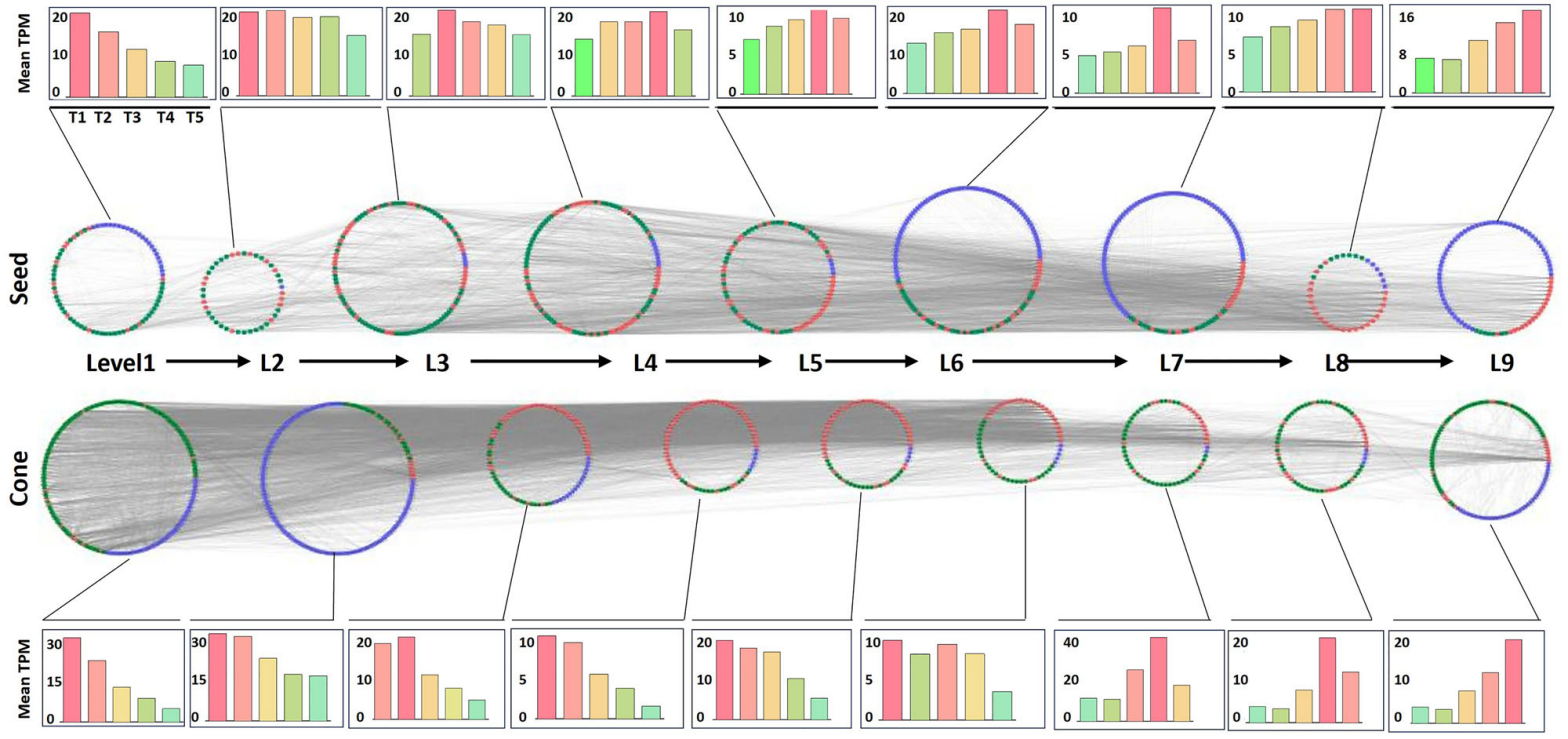
Time-ordered gene co-expression networks (TO–GCN) for *P. massoniana* seeds and cones. The red, green and blue nodes represent TF, gene and lipid metabolites, respectively. Grey lines represent co-expression relationships between two factors. The TPM value of TF gene expression at each level was used to generate the bar charts and heatmap.

Based on KEGG enrichment analysis, DEGs and DAMs containing the term “lipid metabolism” were selected to construct a TO–GCN. Based on expression profiles of transcription factor (TF) genes, a nine-tiered TO–GCN was constructed in seeds and cones (Fig. 5). TO–GCN levels represent the timing of TF gene expression. Lower tiers encompass TFs and non-TFs expressed during early maturation stages, along with DAMs that accumulate during the same period; higher tiers comprise TFs and non-TFs expressed during late maturation, together with DAMs that accumulate at these later stages. Detailed information regarding TO–GCN, including annotation data for TFs, non-TFs, and DAMs, as well as their respective hierarchical levels, have been included in the Supporting Information material (Supplementary Data 8–11).

Among networks, key TF families were revealed (e.g., MYB, AP2, WRKY, bHLH, AUX_IAA, and bZIP), of which MYB, AP2, and WRKY were most abundant in seeds and cones (Supplementary Data 12). DAMs exhibited two different accumulation patterns in cones and seeds, suggesting that seed and cone maturation may be coordinated through opposing lipid metabolic pathways.

### Cone maturation regulatory network revealed by TO–GCN analysis

**Fig. 6:**
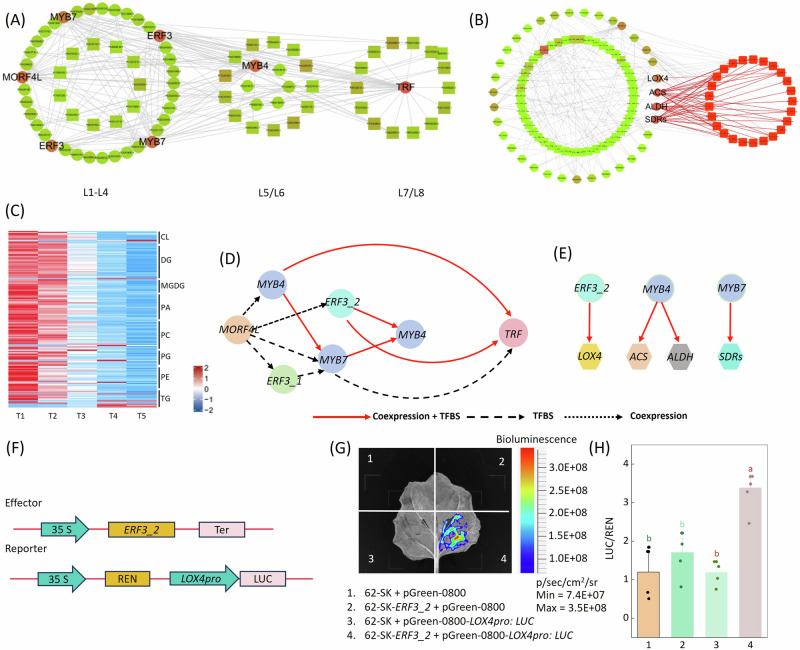
The TO–GCN subnetwork and functional validation of regulators associated with cone maturation. **A** Subnetwork of transcription factors (TFs) associated with cellular senescence. Circular and square nodes represent TFs and genes respectively. The node color indicates the degree of connectivity, which gradually increases from green to red, with darker colors corresponding to larger values. The connecting lines represent the potential co-expression relationships between TFs and genes. **B** Highlighted the potential regulatory factors with the highest connectivity in the subnetwork and the metabolites they regulate. Circular and square nodes represent genes and metabolites, respectively. The node color indicates the degree of connectivity, which gradually increases from green to red, with darker colors corresponding to larger values. **C** Heatmap showing stage-resolved accumulation of metabolites putatively regulated by hub genes. The heatmap depicts the after Z-score normalization across rows, with expression levels indicated by a color gradient ranging from blue to red, representing low to high levels, respectively. **D**, **E** Simplified schematic of predicted TF–target relationships. Circles and pentagons represent TFs and genes, respectively. The red line indicates the presence of transcription factor binding motifs (TFBM) within the promoter of the target gene (as indicated by the arrows), and there exists a co-expression relationship. The dashed line signifies the TFBM solely within the target gene promoter. The dotted line denotes the existence of co-expression relationships alone. **F** Schematic of the effector and reporter constructs used for dual-luciferase assays. Effector = 35S::ERF3_2; reporter = LOX4 promoter driving LUC. **G** Representative qualitative luciferase images from transient expression assays. **H** Quantification of reporter activity. The LUC/REN ratio was calculated to determine promoter activity; the control group LUC/REN value was set to 1. Values are mean ± SD from four independent experiments (*n* = 4). Different letters denote statistically significant differences (one-way ANOVA followed by Fisher’s least-significant-difference [LSD] test; *p* < 0.05).

In the cone-specific TO–GCN, many regulatory factors associated with cellular senescence exhibited the highest connectivity across multiple levels. For example, our analysis placed mortality factor 4-like protein (*MORF4L*)—known to be involved in cellular senescence^28^—in the level one (L1). We also identified an ethylene response factor 3 (*ERF3*)—a regulator of ethylene biosynthesis and senescence^29^—in L4, and a telomere repeat-binding factor (*TRF*)—a key factor in maintaining telomere length and replicative senescence^30^,—in L8. Additionally, several TFs from the MYB family were also highly connected, indicating potential regulatory relationships with TFs related to cellular senescence (Fig. 6A; Supplementary Data 13).

A subnetwork constructed based on core TFs identified three lipid oxidation-related genes: aldehyde dehydrogenase (*ALDH*), responsible for clearing lipid peroxidation products^31^; Lipoxygenase 4 (*LOX4*), which catalyzes polyunsaturated FA oxidation^32^; and short-chain dehydrogenase/reductase (*SDRs*), involved in phospholipid homeostasis^33^. Additionally, a gene encoding acetyl-CoA synthetase (*ACS*) was also identified (Fig. 6B; Supplementary Data 13). The regulated GPLs (including PE and PC as core membrane components) exhibited a time-dependent decline in abundance (Fig. 6C). These results indicate that decreased GPL metabolism correlates with, and may contribute to cellular aging, potentially through alterations in membrane fluidity and oxidative stress responses.

Based on co-expression relationships, we cloned the promoter sequences of candidate target genes to predict their interactions (Supplementary Fig 4). Results indicate that *MYB4* (L4) may interact with *MYB7* (L5) and *TRF* (L8), and *MYB7* (L5) may interact with *MYB4* (L6) in turn; and both *ERF3_2* (L4) and *MYB4* (L4) may interact with *MYB7* (L5) (Fig. 6D). Similarly, we inferred interactions between TFs and core lipid metabolism genes. Results suggest that expression of *LOX4* is likely regulated by *ERF3*_2; *MYB7* may modulate the expression of *ACS* and *ALDH*; and *MYB4* may regulate expression of *SDRs* (Fig. 6E). Dual-luciferase assays further confirmed the in vivo interaction between *ERF3*_2 and pro-*LOX4*. These results indicate that co-expression of *ERF3*_2 with pro-*LOX4* significantly increased fluorescence intensity compared with the control (Fig. 6F-H).

### Seed maturation regulatory network revealed by TO–GCN analysis

**Fig. 7:**
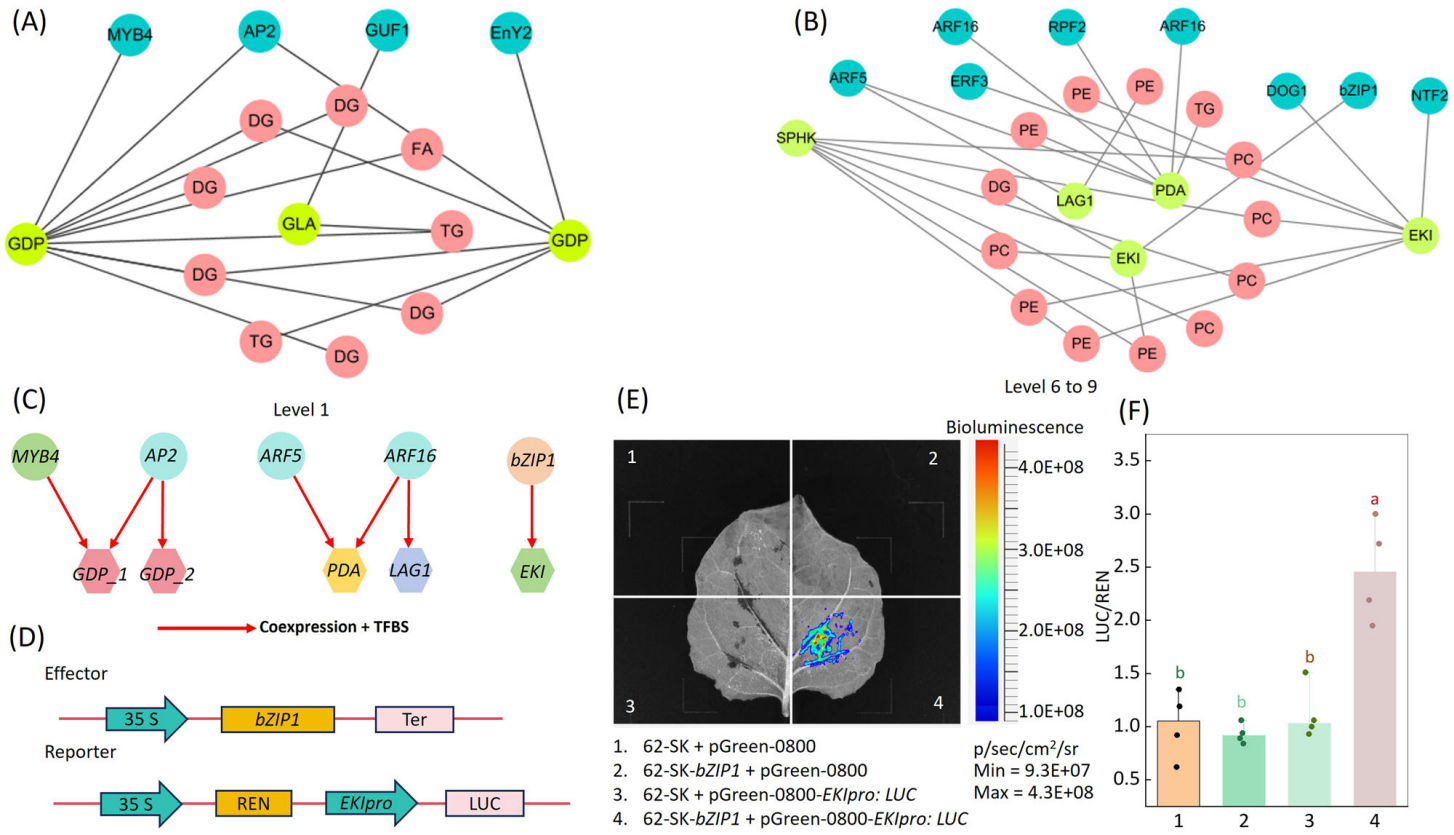
The TO–GCN subnetwork and functional validation of regulators associated with seed maturation. Potential regulatory subnetwork in the early (**A**) and late (**B**) stages of seed maturation. The blue, yellow and red nodes represent TF, gene and lipid metabolites, respectively, and each grey line represents a co-expression relationship between two factors. **C** Simplified schematic of predicted TF–target relationships. Circles and pentagons represent TFs and genes, respectively. The red line indicates the presence of TFBM within the promoter of the target gene (as indicated by the arrows), and there exists a co-expression relationship. **D** Schematic of the effector and reporter constructs used for dual-luciferase assays. Effector = 35S::bZIP1; reporter = EKI promoter driving LUC. **E** Representative qualitative luciferase images from transient expression assays. **F** Quantification of reporter activity. The LUC/REN ratio was calculated to determine promoter activity; the control group LUC/REN value was set to 1. Values are mean ± SD from four independent experiments (*n* = 4). Different letters denote statistically significant differences (one-way ANOVA followed by Fisher’s least-significant-difference [LSD] test; *p* < 0.05).

Based on the seed-specific TO–GCN, to construct a subnetwork associated with seed maturation, we identified genes showing co-expression relationships with 49 key DAMs (Fig. 2A) and potential TFs regulating these genes. This subnetwork was mostly enriched during early (Level 1; T1 and T2) and late maturation stages (Levels 6–9; T5). The early maturation subnetwork comprised two glycerophosphoryl diester phosphodiesterase (*GDP*) genes and one α-galactosidase (*GLA*) gene, along with their upstream regulatory TFs (Fig. 7A; Supplementary Data 14). Annotation of TFs within the subnetwork revealed these genes to be mostly regulated by *MYB4*, *ARF16*, *GUF1*, and *EnY2*. Notably, GO enrichment analysis demonstrated significant overrepresentation of biological processes, including diacylglycerol acyltransferase activity, triglyceride biosynthesis, and triglyceride metabolic processes (*p* < 0.01) (Supplementary Fig 5). These findings suggest that dynamic glycerol metabolism is a prominent feature during early stages of seed maturation.

In late-maturation stages, several genes encoding sphingosine kinase (*SPHK*), sphingosine N-acyltransferase (*LAG1*), phospholipid diacylglycerol acyltransferase (*PDA*), and *EKI* were identified (Fig. 7B; Supplementary Data 14). This demonstrates significant enrichment in membrane lipid biosynthesis and metabolism processes (Supplementary Fig 5) and suggests that, during this phase, reorganization of membrane lipid components and regulation of dynamic equilibrium may represent key processes. We also report various hormone response factors, including auxin response factors (which regulate auxin-dependent gene expression) and *ERFs* (which mediate ethylene signal transduction), to exhibit co-expression relationships with these genes (Fig. 7B; Supplementary Data 14), implying a potential synergistic relationship between lipid metabolism and hormone signaling pathways (auxin/ethylene) during seed maturation. Predicted interactions among these factors further support the notion that lipid metabolism and hormone signaling pathways may act synergistically during seed maturation (Fig. 7C). We selected the pair between *bZIP1* and *EKI* for experimental validation. Dual-luciferase reporter assays confirmed their in vivo interaction (Fig. 7D-F).

### Verifying expression of key genes by qRT‒PCR

qRT-PCR was performed to validate the expression patterns of key genes that were potentially involved in cone and seed maturation. Results confirmed that, within the regulatory network associated with cone maturation, 16 genes (e.g. *MORF4L*, *ERF3*, and *ACS*) exhibited expression profiles consistent with qRT–PCR data. Similarly, for seed maturation, the expression patterns of 20 important genes (e.g., *ARF16*, *bZIP1*, *PDA*, and *GDP*) were also validated (Supplementary Fig. 6). To further substantiate these findings, we performed qRT-PCR validation of key regulatory genes using samples with distinct, independent genetic backgrounds. The results showed that the expression patterns of the core regulatory genes for both cone and seed maturation remained largely consistent despite differences in genetic background (Supplementary Fig. 7).

**Fig. 8:**
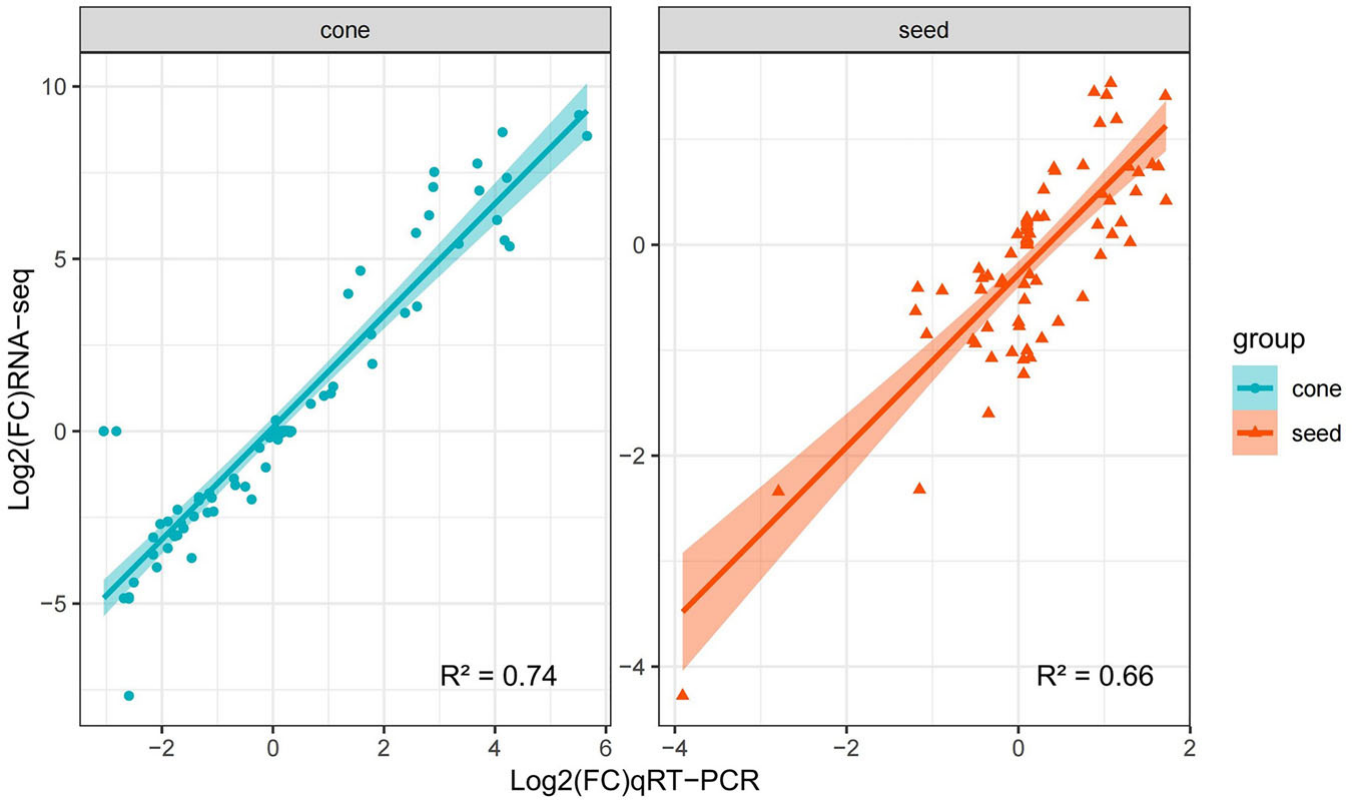
Concordance between RNA-seq and qRT-PCR measurements for core maturation-related genes. Scatter plots compare log₂-transformed fold changes (log₂[FC]) in gene expression measured by RNA-seq (y-axis) and by qRT-PCR (x-axis) for 36 core maturation-related genes: 16 genes from cone and 20 genes from seed maturation, respectively, across five developmental time points. RNA-seq fold changes were calculated from TPM values; qRT-PCR fold changes were calculated by the 2^-^^ΔΔ^^Ct^ method and normalized to an internal reference gene. qRT-PCR data shown are the mean of three technical replicates for each biological replicate; biological replicates, *n *= 3. Solid lines show the linear regression fit for each tissue.

Additionally, the fold change in TPM values from RNA-seq and the relative expression levels from qRT–PCR were log_2_-transformed and subjected to linear regression analysis. Coefficients of determination (R²) were 0.74 for cone and 0.66 for seeds (Fig. 8), indicating a strong correlation between the RNA-seq and qRT-PCR results.

## Discussion

Because the relationship between cone color change and seed maturity varies considerably across conifer species, it is important to establish species-specific harvesting criteria. In some species (e.g., European larch), the development of markedly red cones signals the optimal seed harvest time^27^. In contrast, we report that in *P. massoniana*, a transition from green to brown (browning) is a more reliable visual indicator. Seed germination capacity increases significantly during the browning phase and stabilizes thereafter. This pattern aligns with the synchronous enhancement of G_p_ and G_i_ (Fig. 1D). This suggests that, as for other species where specific color stages correspond to physiological peaks, the browning phase in *P. massoniana* cones marks a window where endosperm nutrition and enzyme activity become optimal for germination^21,23^. Interestingly, while germination parameters plateaued after browning, V_i_ continued to rise, potentially because of ongoing synthesis of internal protective substances such as lipids and antioxidants^8^—a process also noted in the maturation of other oleaginous seeds^34^. Therefore, while cone browning provides a clear visual cue for the onset of high viability, cone cracking might represent a subsequent, and possibly more reliable indicator to obtain seeds at peak vigor.

Our lipidomic profiling revealed glycerolipid and GPL metabolites to predominantly accumulate during the early maturation stages of both cones and seeds (Fig. 2). Notably, the levels of specific polyunsaturated fatty acids (PUFAs), including LA and ALA, increased significantly in later stages. Beyond their acknowledged nutritional value^35,36^, the accumulation of these PUFAs is critically linked to the core physiological processes of maturation^8,11^. In seeds, LA and ALA are key components of storage lipids and membrane GPLs, contributing to energy reserve composition and membrane integrity, which are fundamental to the establishment of germination capacity and vigor^37^. Conversely, in cones, the data suggest that the metabolism of these PUFAs may be channeled towards pathways associated with senescence, potentially through the production of oxylipins or other bioactive molecules that influence aging processes^17^.

A clear divergence in lipid metabolism was apparent between cones and seeds during maturation. In cones, TG levels increased with maturation, whereas GPLs and free FA trended downward, indicating a metabolic shift toward energy storage rather than membrane maintenance. This phenomenon represents a characteristic feature of aging tissues^18,38,39^. In seeds, by contrast, the accumulation of TGs and DGs correlated positively with germination parameters, underscoring their role as carbon and energy reserves^7^. Concurrently, seed vigor was closely associated with specific phospholipids (PC, PE, PG) that are essential for membrane system construction and stability^40^. These findings suggest that pine seed vigor relies on the synergistic effects of TG/DG-mediated energy supply and the involvement of GPLs in membrane system stability. Noteworthy is that ultrasonic treatment can induce a rejuvenation effect in aged *P. tabuliformis* seeds, an outcome attributed to the remodeling of specific membrane lipid classes, such as sphingolipids^10^. This suggests that targeted membrane lipid remodeling, whether induced by an external stimulus or an internal developmental program, is a cornerstone of seed vigor in pines. Furthermore, despite differences in the overall lipid accumulation trends between *P. massoniana* and *Pinus armandii*, GPL metabolism is a central pathway during seed maturation in each^8^. This suggests that the regulation of membrane lipid composition constitutes a conserved biological process in the seed maturation of *Pinus* species.

Cone maturation is closely related to seed dispersal, which simultaneously signifies the gradual senescence and functional decline of associated organizational organs^5,14,19^. We report cones to gradually mature and crack (Fig. 1A). This genetically controlled process is manifested by gradual upregulation of senescence-related regulatory factors (Fig. 6A). While the roles of these factors in leaf senescence or model plant aging are emerging, their coordinated action in cone maturation, particularly in gymnosperms, remains less charted. Specifically, *MORF4L2*, influencing cell proliferation and senescence balance via NuA4 complex regulation^28^, along with ERF as the ethylene signaling pathway core regulator^29^, MYB TFs, and the telomere-binding protein TRF, appear to form a regulatory network unique to cone senescence (Fig. 6B). This network potentially governs the expression of genes involved in lipid oxidation (e.g., *LOX4*, *ALDH*, and *SDRs*), and shows regulatory relationships with GPLs like PE, PC, and PG, whose levels decline during maturation (Fig. 6C, D). Whereas previous research has broadly associated GPL reduction and membrane lipid metabolism imbalance with aging in various plant tissues and neuronal systems^7,18,41,42^, we directly link this phenomenon to cone maturation and cracking. Based on these findings, we suggest that aging factor–may mediated imbalance in membrane lipid metabolism is an important mechanism driving cone maturation and cracking.

The regulatory networks we identify have parallels in both conifer and dicot systems, suggesting conserved mechanisms. Our TO–GCN analysis delineated two sequential regulatory phases governing lipid metabolism during seed maturation in *P. massoniana*. In the early phase, genes such as *GDP* and *GLA* formed a core module associated with TG and DG metabolism. Regulatory network inference suggests that TFs including *MYB4* and *AP2* may act upstream of these genes. This hypothesis is supported by functional studies in other species. For instance, R2R3-MYB TFs in the green alga *Chromochloris zofingiensis* coordinately regulate multiple steps in lipid assembly^43^. In *Arabidopsis thaliana*, the AP2 family member *WRINKLED1* (*WRI1*) binds to conserved AW-box sequences in upstream regions of FA synthesis genes^44–46^.

In the late stages of seed maturation within the co-expression network, the core lipid metabolism genes (e.g., *SPHK*, *EKI*, *PDA*, and *LAG1*) have been identified to possess homologous genes in *A. thaliana*. These enzymes play important roles in seed development. For example, over- and silenced expression of *SPHK* in *A. thaliana* leads to delayed and accelerated germination of mature seeds, respectively^47^. Similarly, overexpression of *EKI* increased PC and TG contents in mature *A. thaliana* seeds, and knockout of *EKI* caused embryonic developmental defects^48^. Within the constructed network, hormone response factors (including ARFs and ERFs) exhibit potential regulatory relationships with these genes. Similar regulatory relationships have been identified in other studies. For instance, in rice, ARFs promote the coordinated accumulation of starch and lipids in the endosperm by directly activating genes such as *OsBUL1*^49^. Similarly, ethylene signaling synergizes with free FAs released by phospholipase A2 to provide energy through β-oxidation to activate the jasmonic acid pathway, delaying lipid peroxidation during maturation^50,51^.

**Fig. 9:**
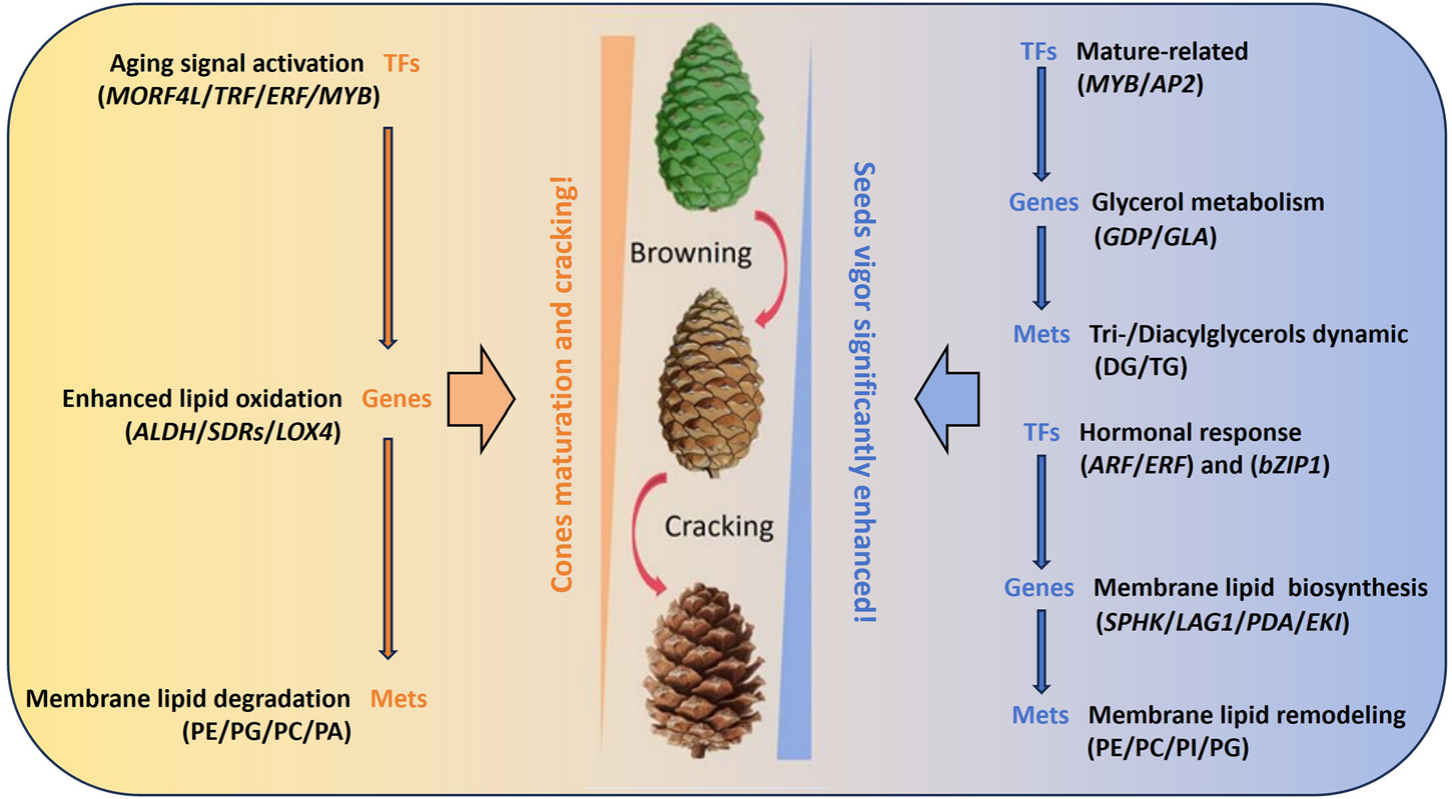
Hypothetical model for lipid metabolism regulating cone and seed maturation in *P. massoniana.* Transcription factors (TFs) and gene. *MORF4L*: Mortality factor 4-like; *ERF*: Ethylene response factor; *TRF*: Telomere repeat-binding factor; *ALDH*: Aldehyde dehydrogenase; *SDRs*: Short-chain dehydrogenase/reductase; *LOX4:* Lipoxygenase 4; *GDP*: glycerophosphoryl diester phosphodiesterase; *GLA*: α-galactosidase; *ARF*: Auxin response factor; *SPHK*: Sphingosine kinase; *LAG1*: Sphingosine N-acyltransferase; *PDA*: Phospholipid diacylglycerol acyltransferase; *EKI*: ethanolamine kinasease. Metabolites (Mets). PE: Phosphatidylethanolamine; PG: Glycerophosphatidylglycerol; PC: Phosphatidylcholine; PA: Phosphatidic acid; PI: phosphoinositol; DG: Diglycerides; TG: Triacylglycerols.

In conclusion, we determine that the optimal time to harvest *P. massoniana* seeds is at the cone cracking stage to acquire the most vigorous seeds. Cone senescence is characterized by triglyceride accumulation and GPL degradation, while seed maturation is associated with coordinated shifts in lipid classes, with TGs/DGs being positively associated with germination traits and GPLs with vigor-related traits. We propose a model in which lipid metabolism is coordinated with these processes through TFs (*MYB, AP2, bZIP, ARF*, and *ERF*) and core genes (*GDP, GLA, LAG1, PDA, EKI, SPHK*), associated with the accumulation of TG/DG (germination) and PE/PC/PI/PG (vigor). Conversely, senescence-associated factors (*MORF4L, ERF, TRF*, and *MYB*) are co-expressed with dehydrogenases (*ALDH, SDRs, LOX4*), coinciding with the disruption of membrane lipid homeostasis (PE/PG/PC/PA) during cone aging and cracking (Fig. 9). Based on this, we establish a mechanistic framework for determining the optimal harvest timing of *P. massoniana* seeds, and elucidate the molecular drivers of lipid dynamics during cone and seed maturation. Future studies could leverage these findings to refine seed-collection protocols and enhance seed quality in pines.

## Methods

### Plant materials

Plant materials were collected from early November to the end of December from a *P. massoniana* clonal seed orchard in a state-owned forest in Huangping County, Guizhou Province, China. Four ramets of the same clone, exhibiting similar growth status, were selected for sampling and used as independent biological replicates (n = 4). Cones were harvested from the same healthy branch at five developmental stages: November 1 (T1), November 16 (T2), December 1 (T3), December 16 (T4), and December 31 (T5); three cones were collected per sampling event from each of the four trees. The cones—the development of which began in March of the previous year—were approximately 20–22 months old when collected.

One cone from each sampling event was dissected to extract seeds. Ovuliferous scales (hereinafter referred to as “scales”) were carefully separated from the cone axis. Given that scales constitute the major structural and physiological component of the cone^5,12,13^, these scale samples were regarded as representative tissues for cone-level analyses. Accordingly, “cone” is used throughout the Results and Discussion sections as well as in subsequent sections of Methods when the RNA-seq and lipidomics experiments are being described or discussed. Immediately frozen in liquid nitrogen, and stored at –80 °C for later transcriptomic and metabolomic sequencing. The remaining two cones were transported to the laboratory for phenotypic evaluation and further analysis.

### Phenotypic and seed germination assessment

Using a fixed-point, fixed-light-source photographic device, fresh cones were photographed, and a CIELAB color model of the images was obtained using Adobe Photoshop 2023 (Adobe Inc., San Jose, CA, USA). Cones were dissected, and healthy seeds were selected. Seed viability was evaluated using the 2,3,5-triphenyltetrazolium chloride (TTC) reduction assay^52^. Briefly, seeds were first imbibed in distilled water to facilitate enzymatic activity. Subsequently, the seed coats were carefully removed, and the embryos were excised. The excised embryos were then immersed in a 1.0% (w/v) TTC solution prepared in phosphate buffer (pH 7.0) and incubated in darkness at 30 °C for 4–6 h. Following incubation, the TTC solution was drained, and the embryos were rinsed with distilled water. Viable embryos, possessing active dehydrogenase enzymes, reduce the colorless TTC to insoluble, red formazan, resulting in distinct crimson staining.

Subsequently, germination rate (G_r_), germination potential (G_p_), germination index (G_i_), and vigor index (V_i_) were determined pursuant to GB/T 2772-1999 standards^53^ according to the following formulas:

$${G}_{r}( \% )=n/N\times 100 \%$$

$${G}_{p}( \% )={n}_{p}/N\times 100 \%$$

$${G}_{i}=\,\sum ({G}_{t}/{D}_{t})$$

$${V}_{i}=\,\sum ({G}_{t}/{D}_{t})\,\times L$$

where N and n represent the total number of seeds and number of germinated seeds, respectively; n_p_ represents the peak number of germinations observed on a single day; G_t_ is the number of germinated seeds on the nth day after the start of germination, and D_t_ is the corresponding number of germination days; and L represents the seedling growth potential, expressed as average embryonic root length.

In brief, these healthy seeds were disinfected with a 0.5% potassium permanganate solution, then rinsed multiple times with deionized water, and then evenly placed on sterilized double-layer filter paper within Petri dishes, to which 10 mL of distilled water was added to each. Petri dishes were placed in a constant temperature illuminated incubator at 25 °C (light intensity 6000 lux) for germination experiments.

From day one, when seeds were hydrated, the germination was monitored daily. Number of germinated seeds were recorded, and embryonic root length was measured, using the criterion of 1 mm of radicle protrusion from the seed coat. The germination experiment concluded when no new seeds germinated after three consecutive days. Similarly, embryonic root growth was considered complete when the growth measurement stabilized, at which point the root-elongation experiment was terminated. To strengthen conclusions, an additional sampling was performed from early November to the end of December 2025. Four individual trees from each of two distinct *P. massoniana* seed orchards in a state-owned forest in Huang Ping and Du Yun County served as biological replicates for comparative analysis.

### RNA-Seq and functional annotation

Total RNA from cones and seeds was extracted using the RNAprep Pure Plant Kit (TIANGEN, China). RNA quality was assessed through NanoDrop 2000 spectrophotometry (Thermo Scientific, USA) and agarose gel electrophoresis. Qualified RNA samples were used for cDNA library construction. mRNA was enriched from 1 μg of total RNA using oligo (dT)-attached magnetic beads. The SMARTer PCR cDNA Synthesis Kit (Takara Holdings Inc., Japan) was used for subsequent library construction. The resulting cDNA library was PCR amplified. After cDNA library qualification with a QSEP400 (BiOptic Inc., China). The cDNA library was sequenced on an Illumina NovaSeq 6000 system by Novogene Corporation (Beijing, China).

Raw sequencing data were processed through a quality control pipeline. Fastp (version 0.23.2) was employed for read filtering and trimming with the following parameters: removal of adapter sequences, trimming of low-quality bases (Phred quality score < Q20) at read ends, filtering reads with a length less than 36 bp after trimming, and sliding window trimming (window size: 4 bp, average quality required: Q20). After this filtering step, high-quality clean reads were retained for downstream analysis.

Transcript abundance was quantified from the clean paired-end reads using Kallisto (version 0.46.2). First, a transcriptome index was built from the reference transcriptome sequences of *Pinus tabuliformis* using the kallisto index command with a k-mer length of 31^54^. Subsequently, read quantification was performed using the kallisto quant function. The quantification step produced estimated transcript counts and transcripts per million (TPM) values for each sample^2^, which were compiled into a gene-level expression matrix for subsequent differential expression analysis.

Differentially expressed genes (DEGs) were identified using the DESeq2 package in R version 3.6.1, with genes exhibiting |log₂FC | ≥ 1 and an FDR (adjusted *p*-value) < 0.01 classified as DEGs. To visualize the global differences in gene expression profiles across different maturation stages, Principal Co-ordinate Analysis (PCoA) was performed. This analysis was conducted based on the transcript TPM values of all DEGs. The analysis used the Bray-Curtis dissimilarity matrix to calculate the distance between samples. The PCoA was carried out using the R package ‘ape’ (version 5.0). Gene expression dynamics were analyzed using the Short Time-series Expression Miner (STEM) software (v1.3.11)^55^. Clustering analysis of the DEGs, Gene Ontology (GO) and Kyoto Encyclopedia of Genes and Genomes (KEGG) pathway enrichment analyses were performed with DEGs using the OmicShare biological platform (http://www.omicshare.com).

### Lipidomic analysis by LC-MS

Seeds and cones that had been kept in −80 °C were ground into fine powder in liquid nitrogen using a SPEX 6870 Freezer/Mill (SPEX SamplePrep, Metuchen, NJ, USA). The ground powder was subsequently lyophilized in a vacuum-freeze dryer. Forty milligrams (mg) of lyophilized powder of each sample were then taken for metabolite extraction in methanol extraction buffer containing recovery chemical standards. After centrifugation to remove protein pellet, the supernatant containing extracted metabolites was transferred into aliquots for LC-MS and LC-MS/MS analyses.

To achieve comprehensive coverage of lipids with diverse chemical properties, the LC-MS analysis was performed in both positive and negative electrospray ionization modes. Employed an ACQUITY CSH C18 column (2.1 × 100 mm, 1.7 µm) at 55 °C with 400 μL/min flow, using mobile phases A (acetonitrile/water 60:40) and B (isopropanol/acetonitrile 90:10) containing 10 mM ammonium formate/0.1% formic acid, under a 20.1 min gradient (40% → 70% B in 12.1 min, 99% B for 6 min, re-equilibration at 40% B), with lyophilized samples reconstituted in 500 μL isopropanol/acetonitrile/water (2:1:1) and analyzed triplicate on a Waters QTOFMS (SYNAPT G1 HDMS) in ±ESI mode (desolvation gas 800 L/h at 400 °C, capillary/cone voltages 3000/35 V), using leucine enkephalin ([M + H]⁺ 556.2771 Da ESI + ; [M-H]⁻ 554.2615 Da ESI − ) for lock mass calibration.

The raw data after sample analysis were pre-processed using Lipidsearch data processing software. First, a simple screening was carried out based on parameters such as retention time and mass-to-charge ratio. Peak extraction was initially performed according to the set ppm, signal-to-noise ratio (S/N), adduct ions and other information. Then, peak alignment was conducted for different samples according to the retention time deviation and mass deviation (Part per million, ppm). Finally, the qualitative and quantitative results of lipid substances were obtained. The screening of differential lipid compounds primarily relies on VIP (Variable Importance in Projection), FC (Fold Change), and *P*-value, with thresholds set as VIP > 1.0, FC > 1.2 and *P*-value < 0.01. To visualize the global differences in lipidomic profiles across different maturation stages, Principal Component Analysis (PCA) was performed.

### Time-ordered gene co-expression network (TO–GCN)

Following Chang et al^56^, we constructed TO–GCNs to analyze time-course transcriptomes across cone and seed tissues. Pearson correlation coefficients (PCC) were computed for all gene pairs (or gene–metabolite pairs). A PCC > 0.84 represents a significant positive co-expression relationship, that <–0.84, significant negative relationship, and values between –0.84 and 0.84, not significant relationships^57^. We focused on tissue-specific positive regulatory networks, namely the cone- and the seed- specific TO**–**GCN. Genes with peak expression at T1 and downregulated thereafter were selected as bait genes, and the TO–GCN hierarchical structure was generated using a breadth-first search algorithm. The source code, documentation, and test datasets utilized for network analysis are available at https://github.com/petitmingchang/TO–GCN^31^. The regulatory network was visualized using Cytoscape (v3.7.1).

### Predicting transcription factor (TF) binding sites and their target genes

To predict the binding sites of TF for *P. massoniana*, known TF binding sites (Position Weight Matrices; PWMs) of *Arabidopsis* TF genes were sourced from the JASPAR TF database^56^. Because TFs with similar DNA binding domains (DBDs) exhibit analogous preferences for DNA sequences, we used the DBDs of *P. massoniana* TFs to identify homologous DBDs in *Arabidopsis* with known TF-TF binding site pairs. Over-represented motifs (PWMs) were identified, and we regarded qualifying PWMs (relative score > 0.8) as putative TF binding motifs (TFBMs).

We adopted a homology‑based cloning approach to obtain the promoter sequences of target genes. Briefly, we first extracted the promoter regions (∼2500 bp upstream of the transcription start site) of candidate genes from the closely related *P. tabuliformis* genome. These sequences were used to design gene‑specific primers (Supplementary Data 15), which were then used to amplify the corresponding promoter regions from *P. massoniana* genomic DNA. Amplified products were cloned and validated using Sanger sequencing. Resulting *P. massoniana* promoter sequences were then used for interaction analysis. Extracted promoter sequences of all genes were scanned using putative TFBMs to identify significant binding sites. A gene was predicted to be a potential target of a specific TF if its promoter contained ≥1 significant binding site (based on the PWM relative score) for that TF’s motif.

### Observing and quantifying luciferase activity

To validate the interactions within the lipid regulatory network, the promoter sequence of lipoxygenase 4 (*LOX4*) and *ethanolamine kinasease (EKI)* was cloned into the pGreenII0800-LUC vector to fuse with the luciferase gene as the reporter construct (driven by T7 promoter), while the full-length CDS of TFs (*ERF3* and *bZIP1*) was inserted into the pGreenII 62-SK vector as the effector construct (driven by 35S promoter). Recombinant plasmids of effectors and reporters, along with empty vector controls, were transformed into Agrobacterium GV3101. Both control and experimental groups were injected into *Nicotiana benthamiana* leaves (OD600 = 0.5). Infected plants were then placed in darkness for 48 h, after which infected leaves were harvested and observed using a Tanon ABL-X5 (Tanon, China). To quantify firefly luciferase (LUC) and rellina luciferase (REN) activity, a *TransDetect*® Double-Luciferase Reporter Assay Kit (Trans, China) was used. The LUC/REN ratio was calculated to determine promoter activity; the control group LUC/REN value was set to 1.

### Quantitative real-time PCR validation

To validate expression patterns of key genes within the lipid regulatory network, fluorescent primers were designed based on the CDSs of 36 key genes using Primer Premier 5.0 (Supplementary Data 15). Three biological and three technical replicates of detection were performed on a CFX Connect Real-Time PCR Detection System (Bio-Rad, USA) using SYBR Green Supermix (Bio-Rad, USA). The reaction system contained 5 μl of Green Supermix, 0.25 μl of upstream and downstream primers, 1 μl of template cDNA, and 3.5 μl of ddH_2_O. The reaction procedure includes a pre-denaturation step at 95 °C for 30 s, followed by 40 amplification cycles (95 °C for 10 s and 60 °C for 30 s). The relative expression levels of the genes were calculated using the 2^-ΔΔCt^ method, with *Ubiquitin C* (*UBC*) gene and *18S rRNA* gene of *P. massoniana* serving as internal references^2^.

### Statistics and reproducibility

Physiological data were analyzed using SPSS software (version 26.0) and expressed as means ± standard errors. All datasets were first tested for normality using Shapiro–Wilk tests. For parametric data, group comparisons were performed using one-way analysis of variance followed by Fisher’s Least Significant Difference test for multiple comparisons. Statistical significance was defined as *p* < 0.05. Sample sizes ranged from 3 to 4 independent biological replicates per condition, depending on the experiment. Specifically, four ramets per clone (*n* = 4) were used for transcriptomic and lipidomic analyses; four cones per time point (*n* = 4) were used for phenotypic and seed germination assessments, with an additional validation set comprising four trees from each of two orchards (*n* = 4 per orchard). For dual‑luciferase reporter assays, four biological replicates were used per construct (*n* = 4). For quantitative real‑time PCR validation, three biological replicates and three technical replicates were performed (*n* = 3). For heatmap visualization, the data matrix was subjected to Z-score normalization across rows to standardize expression values for each gene relative to its mean and standard deviation.

### Reporting summary

Further information on research design is available in the Nature Portfolio Reporting Summary linked to this article.

## Supplementary information

Transparent Peer Review file

Supplementary Information

Description of Additional Supplementary Files

Dataset 1

Dataset 2

Dataset 3

Dataset 4

Dataset 5

Dataset 6

Dataset 7

Dataset 8

Dataset 9

Dataset 10

Dataset 11

Dataset 12

Dataset 13

Dataset 14

Dataset 15

Dataset 16

Reporting Summary

## Data Availability

The raw sequencing data have been deposited in the Genome Sequence Archive at the National Genomics Data Center and are publicly accessible under accession number GSA: CRA033822 at https://ngdc.cncb.ac.cn/gsa. The lipidomic data have been deposited in the OMIX database under accession number OMIX: OMIX013199 and are also accessible via the same National Genomics Data Center portal. The source data underlying the graphs in the main figures and Supplementary Figs. can be found in Supplementary Data 16. All other data supporting the findings of this study are available from the corresponding author upon reasonable request.
