# Supplementary material for: Lipid metabolism-driven regulatory networks involved in cone senescence and seed maturation in pine (Pinus massoniana)
Source: Commun Biol. 2026 May 18;9:1050. doi: 10.1038/s42003-026-10267-z (PMC13444107; doi:10.1038/s42003-026-10267-z)
Supplement: Supplementary file 1 [file 42003_2026_10267_MOESM1_ESM.pdf]

nature portfolio

Peer Review File

Lipid metabolism-driven regulatory networks involved in cone senescence and seed maturation in pine (Pinus massoniana)

Corresponding Author: Professor Feng Wu

This file contains all reviewer reports in order by version, followed by all author rebuttals in order by version.

Version 0:

Reviewer comments:

Reviewer #1

(Remarks to the Author)
This manuscript investigates lipid metabolism during cone senescence and seed maturation in Pinus massoniana by integrating physiological indicators, lipidomics, and transcriptomics. The study highlights the dynamic reprogramming of lipid metabolism and proposes a regulatory model in which lipid metabolic shifts coordinate cone senescence with seed maturation. The work is innovative, with both theoretical and applied significance for conifer seed biology and forestry breeding. The identification of cone color transition as an indicator of seed maturity and the recommendation of optimal seed harvesting time are of practical relevance.
However, several aspects of data presentation, interpretation, and methodological description require clarification and improvement. In particular, some of the mechanistic conclusions are overly speculative given that they rely largely on correlation analyses and TF binding site predictions without experimental validation. The manuscript would benefit from enhanced rigor in data reporting, clearer figure legends, and a more cautious interpretation of results.

Major Comments
1. The description of seed viability indicators is insufficient. The manuscript states that “germination energy and seed viability were determined pursuant to the standards GB/T 2772-1999,” but this lacks sufficient detail. I recommend explicitly providing the formulas for germination rate, germination potential, germination index, and vigor index, as well as describing how the required parameters were measured. In addition, please provide software information for KEGG and GO enrichment analysis.

2. Figures 4 and 5 contain highly complex information, while Figures 6 and 7 focus only on specific subnetworks. To facilitate understanding and reproducibility, the authors should provide comprehensive supplementary materials, including details of STEM clustering results, TO-GCN outputs (composition of different clusters, gene annotations), and full KEGG/GO enrichment results.

3. The expression levels of 12 DEG candidate genes were validated by qRT-PCR. However, Table S1 should include the full names of these genes. Moreover, since transcriptome analysis typically identifies a large number of DEGs, the rationale for selecting only these 12 genes must be clarified. Were these qRT-PCR validations performed on cones or seeds? And what is the specific relevance of these genes to cone or seed development?

4. Although the dataset is relatively comprehensive and the analyses are well executed, the study has clear limitations. (1) All samples were collected from a single natural site, yet environmental factors (temperature, precipitation, etc.) strongly affect cone and seed development. Ideally, environmental condition data during the sampling period (from local monitoring databases) should be added, and comparative data from other sampling sites are encouraged to improve representativeness. (2) The mechanistic analysis highlights several key regulatory genes and proposes potential regulatory relationships; however, these findings remain largely predictive. While it is understood that functional studies in Pinus massoniana are challenging, the authors should at least validate the expression of these key genes by qRT-PCR. Furthermore, any effort to experimentally confirm the predicted interactions would substantially enhance the robustness and credibility of the conclusions.

5. The mechanistic conclusions are largely derived from correlation analyses and predicted regulatory relationships (e.g., TF binding), without experimental validation. The authors should avoid overly speculative language. The statement that "GPL metabolism promotes cellular aging" （Line 337-339） appears too strong, the current TO-GCN analysis only reflects time-dependent co-expression relationships and does not directly establish causality. For example, in the cone-specific TO-GCN, senescence-associated regulators such as MORF4L (L1), ERF (L4), and TRF (L8) appear at different hierarchical levels, while GPLs show a time-dependent decline. It therefore remains unclear whether the observed decrease in GPLs is a causal driver of senescence, or rather a downstream consequence of TF-mediated regulatory processes. To strengthen this conclusion, the authors should carefully rephrase their interpretation, making it clear that GPL decline is correlated with and potentially contributes to cellular senescence, rather than definitively proving a causal relationship. The authors are encouraged to revise similar issues consistently throughout the manuscript.

6. The authors are encouraged to include a more in-depth comparison with existing studies on other conifer species or crop plants in the Discussion. This would help to further highlight the generality and distinctiveness of their findings.

Minor Comments
• Line 273–274: Table S4 is cited but missing.
• Line 308–310: The text refers to Table S5, but the corresponding information currently appears in Table S4. Please check consistency.
• Line 488–490: The authors claim that CCT and LPCAT are identified as core regulatory modules, but these factors are not shown in Figure 7 or the results section. Supporting data should be provided.
• Figure 6: The resolution of the network diagrams should be improved. In particular, the different line types in Figure 6B are difficult to distinguish.

Language and Style
The manuscript requires careful revision for grammar, tense consistency, and academic style. Examples include:
• Abstract, line 12–13: change to "Given the importance of lipid metabolism in maturation, we integrated physiological, lipidomic, and transcriptomic analyses..."
• Abstract, line 16: "Lipidomics revealed dynamic regulation..."
Line 82: "The plant materials were collected..."
• Line 111: "Employed an ACQUITY CSH C18 column..."
• Line 141–142: "Clustering analysis of the DEGs was conducted using..."
• Line 310–311: The sentence ends with an extra parenthesis, which should be deleted.
• Line 467–468: suggest revising to "...aging factor–mediated imbalance in membrane lipid metabolism is an important mechanism driving cone maturation and cracking."

Conclusion and Recommendation
This manuscript presents a valuable and innovative multi-omics study with potential significance for seed biology and forestry applications. However, substantial revisions are required to improve methodological clarity, data presentation, and cautious interpretation of results. I therefore recommend Major Revision before the manuscript can be considered for publication.

Reviewer #2

(Remarks to the Author)
This study employs physiological, lipidomic, and transcriptomic analyses of Pinus massoniana cones and seeds to systematically uncover the role of lipid metabolism in regulating senescence and germination capacity. The results provide important frontier theoretical foundation for molecular breeding of this species. The study is well-designed, the data are comprehensive, and the scientific value is high. However, I would like to offer the following suggestions for further improvement:

1. In Line 14 of the abstract, "we found seed germination capacity rose..." uses a lowercase "we", which violates the grammatical conventions of academic paper abstracts.

2. In Line 87, the sampling design ("three cones collected at each time point") does not specify whether the "three cones were from the same individual". The lack of clarity on sample independence (e.g., cones from the same individual may lead to repeated genetic backgrounds, affecting experimental reproducibility) undermines the rigor of the study design.

3. The manuscript does not specify the post-hoc test used after ANOVA or whether data normality was assessed. Please clarify the statistical methods used, especially for Figure 1. Furthermore, it is not specified in the heatmaps whether normalization was performed. It is recommended that a description of the relevant method (i.e., details of normalization, if applied) be supplemented in the Materials and Methods section

4. Figures 6 contain multiple subpanels with overlapping information and small labels, reducing readability. Consider simplifying these figures or splitting them into supplementary materials for clarity.

5. There are minor errors (e.g., "pant materials" in line 82). A thorough proofreading is recommended.

6. The statement "Data will be made available on request" is vague. Please specify whether raw sequencing data, lipidomic

data, and code for network analysis have been deposited in public repositories (e.g., NCBI, MetaboLights, GitHub), along with accession numbers if available.

7. It is not detailed in the manuscript how the directionality of co-expression relationships (e.g., whether the regulatory relationships are positive or negative) was determined. It is recommended that the relevant methods for confirming this directionality be supplemented, or more specific algorithmic bases (e.g., the criteria for defining positive/negative co-expression in the co-expression network construction process) be cited.

8. The formatting of references is not consistent (e.g., References 21 and 24). It is recommended that a careful check of the reference formatting be conducted throughout the entire manuscript to ensure uniformity in style

Minor Revision is recommended. Addressing these points will significantly enhance the manuscript's rigor, clarity, and impact.

Reviewer #3

(Remarks to the Author)
This manuscript will likely interest researchers in gymnosperm reproduction and the broader plant developmental biology community. While the relationship between seed maturity and cone development has been studied, this detailed examination of the role and regulation of lipids in seed maturation and cone cracking provides novel insights into the underlying mechanisms of these processes.

There are publications describing very similar approaches to understand lipid accumulation in different pine species e.g., Hao et al., 2025. Food Chemistry, 481, 143895, and Li et al., 2024. Journal of Agricultural and Food Chemistry, 72, 21533-215. These do not investigate the cone component of seed maturation or the control of cracking but are sufficiently relevant to consider raising points of similarity and difference in the discussion.

In some sections of the results, it is unclear whether comments are based on the results obtained in this study or previously published results. A good example is the section starting in ln. 376. My interpretation of this would be this was based on previous work but the fact that the materials and methods describe predicting binding sites of transcription factors and their target genes suggests it in novel information from this work?

The Discussion is perhaps a little long and descriptive and at times and rather too speculative. Given that a substantial component of the discussion covers cone browning and lipidomic profiling it would be useful to capture previous work focused on this.

It might also be useful to note that there is considerable variety in cone and seed maturity across conifer species, particularly the link between colour and seed quality.

Some suggestions for potential improvements:

1. There are several places were linking statements are needed for the content to make sense, e.g., in the abstract (ln. 11) – "Given the importance of lipid metabolism in maturation, integrate physiological, lipidomic, and transcriptomic analyses of Pinus massoniana cones and seeds to construct co-expression networks to elucidate maturation mechanisms." Could perhaps read "Given the importance of lipid metabolism in maturation, we have integrated physiological, lipidomic, and transcriptomic analyses of Pinus massoniana cones and seeds to construct co-expression networks to elucidate maturation mechanisms."
2. Likewise, units are often given without context e.g. the abstract (ln. 16) "seed Vigor peaked at 117.5" does not reveal much without units or a definition of seed vigour. It is perhaps better to leave out units until they appear in the main text with some form of explanation.
3. There appears is some confusion regarding lipids and fats and how these are defined. In the introduction, starting on ln. 39 cone seeds are described as being "rich in unsaturated fatty acids …… their lipid content can exceed 15%. Later, on the same page, ln. 50 it is stated that "fat content in mature seeds can exceed 60%. The commentary around these two statements is a little repetitive as well as contradictory.
4. In the materials and methods, is there a simpler way to express "belonged to the same asexual lineages" (ln. 87)? I am not sure what is being implied here? Are these four trees clones derived from a single parental lineage or is there some other meaning being suggested by lineages?
5. In the materials and methods (ln. 87) it would be useful to define the age of the cones at collection as there is quite a lot of variation in the ages that gymnosperm cones reach maturity.
6. Given the importance of the seed indexes such as germination potential, germination index, and vigour index in the results section, it would be useful if an explanation of these were given in the materials and methods. The reference given, the difficult to obtain standard (ln.102) does not allow assessment of what these indexes show. This makes it difficult to interpret the comments regarding the relevance of changes that occur after key seed factors are optimal (ln 400)
7. The first-time abbreviations are used in the text it would be useful to share what they are e.g., PCoA (ln. 274) and TO-GCN (ln. 303). The full names are given in the figure legends but for ease of understanding it would be nice to make this clear the first time they are read. The use of PCoA confused me as principal component analysis is defined as (PCA) on ln. 225.
8. The gene on ln. 486 should read WRINKLED1 (WRI1) rather than WEINKLED1. There is established knowledge on the

role of this gene that is wider than Arabidopsis. Alternative references such as To et al. (2012) and Tan et al. (2023) would better reflect this.

Some minor typographic errors: In, 82 - pant, In 111- ployed

Reviewer #4

(Remarks to the Author)
Cone maturation is accompanied by a series of physiological changes. This manuscript aims to investigate the changes in lipid composition and gene expression in ovuliferous scales and seeds during cone maturation using transcriptomic and metabolomic analyses, and to reveal potential gene regulatory networks involved in ovuliferous scale cracking and seed maturation. However, the manuscript is written in a rather rough manner. There are issues with figure citations, descriptions of figure results, and the use of abbreviations for specialized terms. In addition, the manuscript needs to provide further evidence to support the potential regulatory relationships between transcription factors and key target genes, rather than inferring them solely from gene co-expression networks. Therefore, the manuscript requires substantial revision. The specific issues identified in the manuscript are as follows:

1. Line 9-31: The abstract largely repeats the description of the results and lacks emphasis on the key points. In addition, the term 'glycerophospholipid' appears with two different spellings, as 'glycerophospholipid' and 'glycerol phospholipids'.

2 .Line 35-43: This paragraph does not align with the main theme of this article. Especially the last sentence : 'Despite this, pine seeds remain underexploited', I don't know what the author intends to convey.

3. Line 44-57: According to this section, lipid biosynthesis plays an important role in seed maturation of gymnosperms. However, the authors should critically discuss the limitations of previous studies. In addition, when citing earlier work, the authors are advised to include the genus or species names to ensure that readers clearly understand the research context.

4. Line 58-66: In this paragraph, no studies are cited that demonstrate a role of lipid biosynthesis and metabolism in cone maturation and dehiscence. The authors should cite relevant studies, if available.

5. Line 67-79: The authors should provide a clear overview of the current status of research on cone and seed maturation mechanisms in Pinus massoniana. In addition, the authors should clarify the limitations of previous studies and explain how this study addresses questions that remain unresolved.

6. Line 89-91: It is unclear whether the term 'scales' mentioned here refers specifically to the ovuliferous scale. Moreover, although scales and seeds were used as experimental materials, the manuscript consistently uses the term 'cone' instead of 'scale' in the results section (e.g., lines 249 and 253). The authors should clarify the intended meaning of 'scales' and maintain consistent terminology throughout the manuscript."

7. Line 137-140: This manuscript uses the genome of Pinus tabuliformis as the reference for mapping the transcriptome data of Pinus massoniana. The authors should report the mapping rate to ensure the reliability and reproducibility of the analyses.

8. Line 159-168: In this section of the manuscript, only the method for predicting transcription factor binding sites is described, while the approach for predicting interactions between transcription factors and their target genes is not explained. How did the authors obtain the promoter sequences of the target genes in Pinus massoniana?

9. Line 170: qRT-PCR validation included only 12 randomly selected genes and did not emphasize key lipid metabolism regulators. Could the authors clarify the selection criteria and consider validating core functional genes to strengthen the conclusions.

10. Line 220: “3076 positive and 2088 negative ion metabolites”, how were those results obtained? The authors do not clarify to which parameter the reported “positive” and “negative” correlation' with metabolite accumulation refer. In addition, ion is any atom or group of atoms that bears one or more positive or negative electrical charges, the metabolites listed in Table S2 are not ion metabolites. The authors should revise the terminology to ensure accuracy and consistency throughout the manuscript.

11. Line 225: Some abbreviations in the manuscript appear only once and are not used thereafter; in such cases, abbreviations are unnecessary (e.g., line 225). In addition, some terms are abbreviated more than once (e.g., line 254 and 235). The authors should thoroughly standardize the use of abbreviations to ensure accuracy and improve readability.

12. Line 228: The term 'differentially expressed metabolites (DEMs)' is used inappropriately. The authors should replace it with 'differentially accumulated metabolites' or 'differential metabolites,' The authors should avoid using the verb 'express' with 'metabolite,' as this usage is inappropriate and may confuse readers.

13. Line 234: 'from T1–T5' should be revised to 'from T1 to T5'.

14. Line 331: The figure citations are incorrect, and moreover, the legend of Figure 6 does not correspond to the figure.

15. Line 357-381: Figure 7 is not cited in sequential order.

16. Line 441-418: The discussion on the nutritional and medicinal roles of LA and ALA, and other compounds is relatively long and somewhat diverges from the core mechanisms addressed in this study. Could the authors better link these points to the core mechanism of seed and cone maturation in P. massoniana.

17. Line 334: In Figure 6B, the lipoxygenase 4 gene is not shown; instead, the ACS gene is displayed. Furthermore, aldehyde dehydrogenase and short-chain dehydrogenase/reductase are mentioned in the text without providing their abbreviations, yet their abbreviations appear directly in Figure 8.

18 Line 427: What is the concept and main evidence supporting 'lipid metabolic reprogramming'? In addition, how does lipid metabolic reprogramming differ from the 'membrane lipid remodeling' mentioned later, and based on the results, how can reprogramming be distinguished from remodeling?

19. Line 372: In Figure 7B, the DGD1 gene is not shown. In addition, the groups of genes described in lines 370–373 and lines 488–490 are inconsistent, and Figure 7B also does not display the CCT and LPCAT genes.

20. Line 480-482 and 500-502: Co-expression networks do not necessarily represent regulatory relationships. Although the methods section states that the JASPAR database was used to predict regulatory interactions between transcription factors and target genes, the key regulatory relationships mentioned in this section were not predicted. Additionally, during cone maturation, the regulatory interactions between key transcription factors (MYB/MORF4L/TRF/ERF) and key genes (ALDH/SDR/ACS) were also not predicted. The authors are advised to include these analyses.

21. Line 529: Based on the results of this study, when is the optimal harvest timing for seeds in Pinus massoniana?

22. Figure 8: Why are the MYB genes absent from the 'Aging signal activation' section in Figure 8? According to the results and discussion sections, MYB genes appear to play an important role in cone senescence.

Version 1:

Reviewer comments:

Reviewer #1

(Remarks to the Author)
In the revised manuscript, the authors have carefully addressed all of the specific concerns and questions raised in my first-round review. The research presents novel and valuable insights into lipid metabolism-mediated regulatory networks during cone senescence and seed maturation in Pinus massoniana, with both theoretical and practical implications for conifer reproductive biology and forest breeding. I consider that the manuscript has met the publication standards and recommend acceptance for publication in Communications Biology.

Reviewer #2

(Remarks to the Author)

Reviewer #3

(Remarks to the Author)
I previously reviewed this manuscript, which describes a detailed analysis of lipid dynamics and their regulation during seed maturation and cone cracking in Pinus massoniana. Through integration of physiological measurements, lipidomics, and transcriptomics, the authors identify substantial reprogramming of lipid metabolism and propose a model linking lipid metabolic shifts with scale senescence and seed maturation. This manuscript provides novel insights of interest to those with both theoretical and applied interest in gymnosperm reproduction and conifer breeding.

I find that the issues I raised in the initial review have been substantially addressed. The manuscript is improved in both readability and clarity. Additional references now provide links to previous work addressing similar scientific questions. The improved figures and added supplementary material give increased understanding of the complex data sets presented.

While the discussion has been refined and is less speculative, there remains a tendency to infer functional significance from correlative observations without supporting experimental evidence. Statements such as "These results reveal lipid classes to regulate seed quality" (paragraph beginning Ln. 375) and "seed maturation requires coordinated shifts in lipid classes: TGs/DGs boost germination and GPLs enhance vigor" (Ln. 631) draw mechanistic conclusions that the data do not provide direct evidence for. As these are correlative findings, the language should be moderated accordingly. Confirming roles for these lipid classes in regulating seed quality or modulating germination traits would require further experimental work beyond the scope of this manuscript.

A couple of minor suggestions:

In the title at Ln. 388, please use "time-ordered co-expression networks" in full before introducing the abbreviation "TO-GCN", as this is its first appearance in the Results section.

The above paragraph – starting at Ln 388 suggests the network includes DAMs expressed at various stages. Is this metabolite accumulation or a measure of expression?

The Materials and Methods section states that ovuliferous scales were extracted from the cone for use in expression analysis and lipid studies, and the term scales is used consistently throughout this section. However, the Results section shifts to referring to cones without explanation.
To avoid confusion, it would be helpful to include a brief clarifying statement that explicitly addresses this terminology change.

Reviewer #4

(Remarks to the Author)
The authors have addressed my previous comments and concerns in their revision.

Open Access This Peer Review File is licensed under a Creative Commons Attribution 4.0 International License, which permits use, sharing, adaptation, distribution and reproduction in any medium or format, as long as you give appropriate credit to the original author(s) and the source, provide a link to the Creative Commons license, and indicate if changes were made.
In cases where reviewers are anonymous, credit should be given to 'Anonymous Referee' and the source.
The images or other third party material in this Peer Review File are included in the article's Creative Commons license, unless indicated otherwise in a credit line to the material. If material is not included in the article's Creative Commons license and your intended use is not permitted by statutory regulation or exceeds the permitted use, you will need to obtain permission directly from the copyright holder.
To view a copy of this license, visit https://creativecommons.org/licenses/by/4.0/

Response to Reviewers' Comments

Manuscript Number: COMMSBIO-25-7147A

"Lipid metabolism-driven regulatory networks coordinate cone senescence and seed maturation in pine"

Reviewer #1

"*This manuscript investigates lipid metabolism during cone senescence and seed maturation in Pinus massoniana by integrating physiological indicators, lipidomics, and transcriptomics. The study highlights the dynamic reprogramming of lipid metabolism and proposes a regulatory model in which lipid metabolic shifts coordinate cone senescence with seed maturation. The work is innovative, with both theoretical and applied significance for conifer seed biology and forestry breeding. The identification of cone color transition as an indicator of seed maturity and the recommendation of optimal seed harvesting time are of practical relevance.*

However, several aspects of data presentation, interpretation, and methodological description require clarification and improvement. In particular, some of the mechanistic conclusions are overly speculative given that they rely largely on correlation analyses and TF binding site predictions without experimental validation. The manuscript would benefit from enhanced rigor in data reporting, clearer figure legends, and a more cautious interpretation of results."

Response: We are thankful for the comprehensive and encouraging evaluation of our work. We would also like to acknowledge your work and dedication invested—it helped us to thoroughly improve the quality of our manuscript. We have addressed the specific points for improvement in detail below.

Please note that the line numbers mentioned in this response refer to the revised version of the manuscript with changes marked.

Major Comments

1."*The description of seed viability indicators is insufficient. The manuscript states that "germination energy and seed viability were determined pursuant to the standards GB/T 2772-1999," but this lacks sufficient detail. I recommend explicitly providing the formulas for germination rate, germination potential, germination index, and vigor*

index, as well as describing how the required parameters were measured. In addition, please provide software information for KEGG and GO enrichment analysis."

Response: Thanks for your comments and advices. We have supplemented the "Materials and methods" section with detailed descriptions of the calculation formulas and measurement procedures for each indicator.

Line 203-211: "Seed viability was evaluated using the 2,3,5-triphenyltetrazolium chloride (TTC) reduction assay. Briefly, seeds were first imbibed in distilled water to facilitate enzymatic activity. Subsequently, the seed coats were carefully removed, and the embryos were excised. The excised embryos were then immersed in a 1.0% (w/v) TTC solution prepared in phosphate buffer (pH 7.0) and incubated in darkness at 30°C for 4-6 hours. Following incubation, the TTC solution was drained, and the embryos were rinsed with distilled water. Viable embryos, possessing active dehydrogenase enzymes, reduce the colorless TTC to insoluble, red formazan, resulting in distinct crimson staining."

Line 212-244: "Subsequently, germination rate (G_r), germination potential (G_p), germination index (G_i), and vigor index (V_i) were determined pursuant to the standards GB/T 2772-1999 standards according to the following formulas:

$$G_r(\%) = {}^{n}/_{N} \times 100\%$$

$$G_p(\%) = {}^{n_p}/_{N} \times 100\%$$

$$G_i = \sum({}^{G_t}/_{D_t})$$

$$V_i = \sum({}^{G_t}/_{D_t}) \times L$$

where N and n represent the total number of seeds and number of germinated seeds, respectively; np represents the peak number of germinations observed on a single day; Gt is the number of germinated seeds on the nth day after the start of germination, and Dt is the corresponding number of germination days; and L represents the seedling growth potential, expressed as average embryonic root length."

In brief …"

Furthermore, we have updated the Materials and Methods section to include detailed information on the software used for KEGG and GO enrichment analyses and have provided the corresponding URLs.

Line 264-267: "Clustering analysis of the DEGs, Gene Ontology (GO) and Kyoto Encyclopedia of Genes and Genomes (KEGG) pathway enrichment analyses were

performed with DEGs using the OmicShare biological platform (http://www.omicshare.com)."

2. "*Figures 4 and 5 contain highly complex information, while Figures 6 and 7 focus only on specific subnetworks. To facilitate understanding and reproducibility, the authors should provide comprehensive supplementary materials, including details of STEM clustering results, TO-GCN outputs (composition of different clusters, gene annotations), and full KEGG/GO enrichment results.*"

Response: Thank you for your constructive comments and suggestions. As suggested, the detailed results of the STEM clustering, including the assignment of each gene and the annotation information for all clustered genes, are provided in **Tables S4** and **S5**. The complete edge list of the TO-GCN network and the gene composition of each module are available in **Tables S9-S12**. Full KEGG and GO enrichment results have been compiled in **Tables S6** and **S7**.

3. "*The expression levels of 12 DEG candidate genes were validated by qRT-PCR. However, Table S1 should include the full names of these genes. Moreover, since transcriptome analysis typically identifies a large number of DEGs, the rationale for selecting only these 12 genes must be clarified. Were these qRT-PCR validations performed on cones or seeds? And what is the specific relevance of these genes to cone or seed development?*"

Response: We sincerely appreciate you for bringing this matter to our attention. The original set of 12 candidate genes was selected as a representative, technical validation panel because these genes showed (i) relatively high expression levels in both cone and seed tissues and (ii) pronounced, stage-specific differential expression in our RNA-seq dataset. These characteristics made them appropriate for a preliminary validation aimed at confirming the reliability of the transcriptome results. Moreover, in order to strengthen the validation of functionally important genes, we have added another 24 key genes identified as core regulators of lipid metabolism by our integrated analysis. Consequently, a total of 36 key genes (16 associated with cone maturation and 20 with seed maturation) were validated by qRT-PCR using RNA extracted separately from cone and seed tissues (**Figure 8** and **Figure S6**). We have also added the rationale for gene selection to the revised manuscript at line 720-721 and provided the full gene names **Table S1**.

In addition, the 36 genes were selected on the basis of their functional relevance to cone or seed development. Genes validated in cones (e.g., *LOX4*, *ALDH*, *ACS*) are implicated in lipid oxidation and senescence-related pathways associated with cone maturation, whereas genes validated in seeds (e.g., *LAG1*, *PDA*, *SPHK*) encode enzymes central to storage-lipid biosynthesis, membrane-lipid remodelling and sphingolipid metabolism—processes that are important for seed maturation and vigour.

To document these additions, we have added a new Results subsection entitled "Validation of key gene expression by qRT-PCR". The expression patterns of the 36 genes in cones and seeds are shown in **Figure 8** and **Figure S6**, and are described in the revised manuscript at line 720-735.

Comment 4: "*Although the dataset is relatively comprehensive and the analyses are well executed, the study has clear limitations. (1) All samples were collected from a single natural site, yet environmental factors (temperature, precipitation, etc.) strongly affect cone and seed development. Ideally, environmental condition data during the sampling period (from local monitoring databases) should be added, and comparative data from other sampling sites are encouraged to improve representativeness. (2) The mechanistic analysis highlights several key regulatory genes and proposes potential regulatory relationships; however, these findings remain largely predictive. While it is understood that functional studies in Pinus massoniana are challenging, the authors should at least validate the expression of these key genes by qRT-PCR. Furthermore, any effort to experimentally confirm the predicted interactions would substantially enhance the robustness and credibility of the conclusions.*"

Response: We sincerely appreciate you for these constructive comments. To address the limitations raised and to improve the representativeness and mechanistic support of our study, we have implemented the following revisions and additional experiments:

1) Environmental data

Monthly temperature and precipitation records covering the entire sampling period were obtained from the local monitoring database and have been added to the manuscript (**Table S2**).

2) Replication at an independent site

To assess generalizability, we repeated sampling and the same analytical pipeline at an independent *P. massoniana* clonal seed orchard in Duyun, Guizhou Province. Comparative analyses of cone colour parameters (L, a, b*) show consistent maturation

trends between the Huangping and Duyun sites (**Figure S1**). Key seed physiological metrics (germination rate Gr, germination potential Gp, germination index Gi) and the vigour index (Vi) exhibited highly similar temporal patterns across both sites; these data are integrated into **Figure S1**. Together, these results indicate that the cone-colour transition and its correlation with seed physiological maturity are repeatable across sites.

3) qRT-PCR validation of predicted regulators

Recognizing the predictive nature of our network analysis, we experimentally validated expression patterns for the 36 key genes and transcription factors nominated by our integrated analysis. qRT-PCR was performed on RNA extracted separately from cone and seed tissues (samples from Huangping). The results agree with the RNA-seq trends and are presented in **Figure S6**. To confirm robustness across environments, we also quantified expression of these genes in cone and seed samples from the independent Duyun site. The concordant expression profiles are presented in **Figure S7**.

4) Experimental confirmation of a predicted interaction

To provide direct experimental support for our predicted regulatory relationships, we selected two representative transcription factor–target gene pairs — one derived from cone maturation process and one from seed — for functional validation by dual-luciferase reporter assays. Both the qualitative luciferase imaging and the quantitative reporter measurements confirmed that the tested TFs activate expression of their predicted target genes. These results are presented in **Figures 6F–H** and **7D–F** of the revised manuscript.

Comment 5: "*The mechanistic conclusions are largely derived from correlation analyses and predicted regulatory relationships (e.g., TF binding), without experimental validation. The authors should avoid overly speculative language. The statement that "GPL metabolism promotes cellular aging"（Line 337-339） appears too strong, the current TO-GCN analysis only reflects time-dependent co-expression relationships and does not directly establish causality. For example, in the conespecific TO-GCN, senescence-associated regulators such as MORF4L (L1), ERF (L4), and TRF (L8) appear at different hierarchical levels, while GPLs show a time-dependent decline. It therefore remains unclear whether the observed decrease in GPLs is a causal driver of senescence, or rather a downstream consequence of TF-mediated regulatory processes. To strengthen this conclusion, the authors should carefully rephrase their*

interpretation, making it clear that GPL decline is correlated with and potentially contributes to cellular senescence, rather than definitively proving a causal relationship. The authors are encouraged to revise similar issues consistently throughout the manuscript."

Response: Thanks for your constructive comments and advices. As suggested, we have conducted a thorough revision throughout the manuscript to replace overly strong and speculative language with more accurate descriptions that reflect the correlative nature of our data.

1) The statement "GPL metabolism promotes cellular aging" (original lines 337-339) has been revised to: "These results indicate that decreased GPL metabolism correlates with, and may contribute to cellular aging, potentially through alterations in membrane fluidity and oxidative stress responses." (lines 614-617 in the revised manuscript).

2) The statement "These findings reveal the early stages of seed maturation to be fundamentally associated with dynamic glycerol metabolism." (original lines 368-369) has been revised to: "These findings suggest that dynamic glycerol metabolism is a prominent feature during early stages of seed maturation." (lines 678-679 in the revised manuscript).

3) The statement "suggests that reorganization of membrane lipid components and regulation of dynamic equilibrium are primary events during this phase." (original lines 374-376) has been revised to: "suggests that reorganization of membrane lipid components and regulation of dynamic equilibrium may represent key processes during this phase." (lines 688-689 in the revised manuscript).

4) The statement "revealing a synergistic regulatory relationship between lipid metabolism and hormone signaling pathways (auxin/ethylene) during seed maturation." (original lines 379-380) has been revised to: "implying a potential synergistic relationship between lipid metabolism and hormone signaling pathways (auxin/ethylene) during seed maturation." (lines 695-697 in the revised manuscript).

5) The statement "Lipid metabolism orchestrates these processes through TFs …, hormone-responsive factors) and core genes …, driving accumulation of … and …. Conversely, senescence-associated factors … activated dehydrogenases …, disrupting membrane lipid homeostasis … to promote cone aging and cracking." (original lines 522-528) has been revised to: "Our analyses propose a model in which lipid metabolism is coordinated with these processes through TFs … and core genes …, associated with the accumulation of … and …. Conversely, senescence-associated factors … are co-

expressed with dehydrogenases …, coinciding with the disruption of membrane lipid homeostasis … during cone aging and cracking" (lines 966-974 in the revised manuscript).

Comment 6: "*The authors are encouraged to include a more in-depth comparison with existing studies on other conifer species or crop plants in the Discussion. This would help to further highlight the generality and distinctiveness of their findings.*"

Response: Thanks for this helpful suggestion. We have substantially expanded the comparative analysis in the revised Discussion by incorporating specific comparisons with both closely related conifer species and distantly related crop plants. The key comparisons now integrated into the manuscript include:

1) We now align our observations on cone color as a maturity indicator with established patterns in other gymnosperms (Lines 747-756), and further discuss how the relationship between color stages and physiological maturity parallels mechanisms in other species (Lines 747-756).

2) We draw a direct functional parallel by citing a study on *P. tabuliformis*, in which membrane-lipid remodelling was linked to improved seed vigour, using this example to emphasize the conserved role of membrane integrity and lipid metabolism in conifer seed quality. (Lines 832-837).

3) We explicitly compare our lipid accumulation trends with those reported in the closely related *P. armandii*, noting that despite differences in overall profiles, glycerophospholipid metabolism is a central, conserved pathway in seed maturation for both species (Lines 837-841).

4) We clarify how prior studies have characterized senescence factors in other tissues or angiosperm systems, but that their coordinated role in cone maturation within gymnosperms has been largely unexplored — a gap our study helps to address. (Lines 845-883).

5) We strengthen our transcriptional regulatory hypotheses by referencing functional studies in model dicots and crops (Lines 894-944).

Minor Comments

Comment: "*Line 273–274: Table S4 is cited but missing. Line 308–310: The text refers to Table S5, but the corresponding information currently appears in Table S4. Please check consistency.*"

Response: We sincerely thanks for your careful reading. In the revised manuscript, we have carefully checked and corrected all citations to supplementary tables throughout the text.

Comment: "*Line 488–490: The authors claim that CCT and LPCAT are identified as core regulatory modules, but these factors are not shown in Figure 7 or the results section. Supporting data should be provided.*"

Response: We apologize for the oversight and appreciate your careful reading. The reference to *CCT* and *LPCAT* was an inadvertent leftover from an earlier analysis and should not have appeared in the revised text. Therefore, in the revised manuscript, we have removed the unsupported claim regarding *CCT* and *LPCAT*. The discussion now focuses exclusively on the core genes robustly supported by our final analysis and presented in **Figure 7**, specifically *PDA*, *LAG1*, *EKI*, and *SPHK*. The descriptions pertaining to the relevant content have been revised throughout the manuscript, as detailed below:

Line 682-686: "In late-maturation stages, … sphingosine kinase (*SPHK*), sphingosine N-acyltransferase (*LAG1*), phospholipid diacylglycerol acyltransferase (*PDA*), and ethanolamine kinase (*EKI*) were identified."

Line 919-921: "In the late stages of seed maturation within the co-expression network, the core lipid metabolism genes (e.g., *SPHK, EKI, PDA*, and *LAG1*) have been identified to ..."

Line 966-969: "We propose a model in which…*LAG1, PDA, EKI, SPHK*), associated with the accumulation of ..."

Comment: "*Figure 6: The resolution of the network diagrams should be improved. In particular, the different line types in Figure 6B are difficult to distinguish.*"

Response: Thanks for your valuable suggestions. To enhance clarity, we have scaled down the ancillary information in **Figure 6** (particularly in **Figure 6A** and **6B**) to emphasize the primary data, and we have relocated the supporting data to **Table S14** for easier reference.

Language and Style

Comment: *"The manuscript requires careful revision for grammar, tense consistency, and academic style. Examples include:*

• Abstract, line 12–13: change to "Given the importance of lipid metabolism in maturation, we integrated physiological, lipidomic, and transcriptomic analyses..."

• Abstract, line 16: "Lipidomics revealed dynamic regulation..."

Line 82: "The plant materials were collected..."

• Line 111: "Employed an ACQUITY CSH C18 column..."

• Line 141–142: "Clustering analysis of the DEGs was conducted using..."

• Line 310–311: The sentence ends with an extra parenthesis, which should be deleted.

• Line 467–468: suggest revising to "...aging factor–mediated imbalance in membrane lipid metabolism is an important mechanism driving cone maturation and cracking."

Response: We sincerely thanks for your careful reading and valuable suggestions. The manuscript has been thoroughly revised to address all points raised, and the changes are incorporated and highlighted in the revised manuscript. In addition, to improve clarity and style, the manuscript has undergone professional English-language editing by native English speakers.

Reviewer #2

"*This study employs physiological, lipidomic, and transcriptomic analyses of Pinus massoniana cones and seeds to systematically uncover the role of lipid metabolism in regulating senescence and germination capacity. The results provide important frontier theoretical foundation for molecular breeding of this species. The study is well-designed, the data are comprehensive, and the scientific value is high. However, I would like to offer the following suggestions for further improvement:*"

Response: We are grateful for your favorable assessment and have addressed their constructive suggestions in detail below.

Comment 1: "*In Line 14 of the abstract, "we found seed germination capacity rose..." uses a lowercase "we", which violates the grammatical conventions of academic paper abstracts.*"

Response: Thank you for pointing out this oversight. The sentence has been amended to "We found seed germination capacity..."

Comment 2: "*In Line 87, the sampling design ("three cones collected at each time point") does not specify whether the "three cones were from the same individual". The lack of clarity on sample independence (e.g., cones from the same individual may lead*

to repeated genetic backgrounds, affecting experimental reproducibility) undermines the rigor of the study design."

Response: We sincerely thanks for raising this important point. We have clarified the sampling design and analytical treatment in the revised manuscript as following:

Line 163–170: "Four ramets of the same clone, exhibiting similar growth status, were selected for sampling and used as independent biological replicates (n = 4). Cones were harvested from the same healthy branch at five developmental stages: November 1 (T1), November 16 (T2), December 1 (T3), December 16 (T4), and December 31 (T5); with three cones were collected per sampling event from each of the four trees"

Initially, we sampled four ramets of a clone to minimize genetic-background variation and thereby resolve developmental- and environment-driven changes with high temporal and physiological resolution. This approach is a widely used and valid strategy when the aim is to characterise ontogenetic trajectories with high precision.

In response to your concern, we established another seed orchard (Duyun, Guizhou Province) and collected an equivalent developmental time series from four ramets of a different clone representing distinct genetic backgrounds. Parallel measurements (cone colour parameters, seed germination metrics Gr/Gp/Gi and vigour index Vi) and qRT-PCR validation of key regulatory genes were performed on these independent samples. The core temporal patterns and the relationships between cone colour and seed physiological maturity were highly consistent between the Huangping and Duyun seed orchards, , with only minor site-specific differences in absolute values. These supporting data are provided in Supplementary **Figure S1** and **Figure S7**, and demonstrate that our main conclusions are robust across different genetic backgrounds and environmental contexts.

Comment 3: "*The manuscript does not specify the post-hoc test used after ANOVA or whether data normality was assessed. Please clarify the statistical methods used, especially for Figure 1. Furthermore, it is not specified in the heatmaps whether normalization was performed. It is recommended that a description of the relevant method (i.e., details of normalization, if applied) be supplemented in the Materials and Methods section*"

Response: Thanks for your helpful comments and advices. We have now fully described the statistical procedures used for Figure 1 (and elsewhere in the manuscript) in the Statistical analysis subsection of Materials and Methods, and have cited this

subsection in the Figure 1 legend. The additional content is as follows:

In the Materials and Methods section, we have added (Line 371-375): "All datasets were first tested for normality using the Shapiro-Wilk test. For parametric data, …followed by Fisher's Least Significant Difference (LSD) test for multiple comparisons. Statistical significance was defined as $p < 0.05$."

In the legend of Figure 1, we have added: (Line 426-428): "Data are presented the mean ± SE (standard deviation) from four biological replicates (n = 4). (with four replicates), Different letters denote statistically significant differences among stages (one-way ANOVA followed by Fisher's least-significant-difference [LSD] test; p < 0.05)"

Furthermore, we have now supplemented a detailed description of the normalization method in the Materials and Methods section and referenced it in the corresponding figure legend. The specific additions are as follows:

In the Materials and Methods section, we added (Line 377-380): "For heatmap visualization, the data matrix was subjected to Z-score normalization ..."

In the legend of Figure 3,4 and 6, we have added: "The heatmap depicts the … after Z-score normalization across rows."

Comment 4: "*Figures 6 contain multiple subpanels with overlapping information and small labels, reducing readability. Consider simplifying these figures or splitting them into supplementary materials for clarity.*"

Response: Thanks for your valuable comments and advices. To improve clarity, we have simplified **Figure 6** (especially panels A and B) by focusing only on the key hub genes central to our study. The additional supporting data, including detailed correlation networks, have been moved to the Supplementary Materials (**Table S14**).

Comment 5: "*There are minor errors (e.g., "pant materials" in line 82). A thorough proofreading is recommended.*"

Response: We sincerely thanks for your carefully reading and for pointing this out. We have conducted a thorough proofreading of the entire manuscript to correct such minor errors and improve the overall language quality.

Comment 6: "*The statement"Data will be made available on request" is vague. Please specify whether raw sequencing data, lipidomic data, and code for network*

analysis have been deposited in public repositories (e.g., NCBI, MetaboLights, GitHub), along with accession numbers if available."

Response: Thanks for your constructive comments. The raw sequencing data and lipidomic data have been deposited in the Genome Sequence Archive (Genomics, Proteomics & Bioinformatics 2025) at the National Genomics Data Center. The data are publicly accessible under accession numbers GSA: CRA033822 and OMIX: OMIX013199 at https://ngdc.cncb.ac.cn/gsa.

Furthermore, the code for network analysis is referenced from Chang et al (2019). We have supplemented the information to the revised manuscript (Line 313-314).

Comment 7: "*It is not detailed in the manuscript how the directionality of co-expression relationships (e.g., whether the regulatory relationships are positive or negative) was determined. It is recommended that the relevant methods for confirming this directionality be supplemented, or more specific algorithmic bases (e.g., the criteria for defining positive/negative co-expression in the co-expression network construction process) be cited.*"

Response: We sincerely thanks for your valuable suggestions. We have now provided explicit methodological details in the revised Methods section (Lines 300–315). The directionality (positive or negative) of co-expression relationships was determined based on the Pearson correlation coefficient (PCC) between gene pairs or gene–metabolite pairs. A PCC > +0.84 was defined as a significant positive co-expression, and a PCC < –0.84 as a significant negative co-expression (Chang et al., 2019).

In our study, our downstream network analysis focused on tissue-specific positive regulatory networks: the cone-specific TO-GCN (Cone+) and the seed-specific TO-GCN (Seed+) (Line 308-310).

Comment 8: "*The formatting of references is not consistent (e.g., References 21 and 24). It is recommended that a careful check of the reference formatting be conducted throughout the entire manuscript to ensure uniformity in style.*"

Response: Thanks for your valuable suggestions. All references, including the specific examples mentioned (References 21 and 24), have been corrected and are now consistent in format.

Reviewer #3

"*This manuscript will likely interest researchers in gymnosperm reproduction and the broader plant developmental biology community. While the relationship between seed maturity and cone development has been studied, this detailed examination of the role and regulation of lipids in seed maturation and cone cracking provides novel insights into the underlying mechanisms of these processes.*"

Response: We sincerely thanks for your recognizing the novel insights of our study and its potential relevance to the broader research community. We have carefully incorporated the suggested comparative analyses and clarifications into the revised manuscript, as detailed below.

Comment: "*There are publications describing very similar approaches to understand lipid accumulation in different pine species e.g., Hao et al., 2025. Food Chemistry, 481, 143895, and Li et al., 2024. Journal of Agricultural and Food Chemistry, 72, 21533-215. These do not investigate the cone component of seed maturation or the control of cracking but are sufficiently relevant to consider raising points of similarity and difference in the discussion.*"

Response: We sincerely thank you for highlighting these relevant studies. We have now incorporated direct comparisons with Hao et al. (2025, Food Chemistry), Li et al. (2024, Journal of Agricultural and Food Chemistry) and other pertinent studies into the revised Discussion to clarify similarities and differences between their findings and ours. Briefly, the main points added are as following:

1) We draw a direct functional parallel by citing a study on *P. tabuliformis*, where ultrasonic treatment-induced membrane lipid remodeling improved seed vigor, underscoring the fundamental importance of membrane lipid homeostasis in conifer seeds (Lines 832-837).

2) We explicitly compare our lipid accumulation trends with those reported in the closely related *P. armandii*, noting that despite differences in overall profiles, glycerophospholipid metabolism is a central, conserved pathway in seed maturation for both species (Lines 837-841).

Comment: "*In some sections of the results, it is unclear whether comments are based on the results obtained in this study or previously published results. A good example is the section starting in ln. 376. My interpretation of this would be this was based on*

previous work but the fact that the materials and methods describe predicting binding sites of transcription factors and their target genes suggests it in novel information from this work?"

Response: Thanks for your constructive comments and suggestions. In the revised manuscript, we have systematically clarified such passages throughout the Results section.

line 376, The original statement that "Various hormone response factors have been identified…" could be misinterpreted as summarizing prior knowledge. We have revised it to explicitly state: "Furthermore, in this study, various hormone response factors, including auxin response factors (which regulate auxin-dependent gene expression) and ERFs (which mediate ethylene signal transduction) exhibit co-expression relationships with these genes (**Figure 7C; Table S15**), implying a potential synergistic relationship between lipid metabolism and hormone signaling pathways (auxin/ethylene) during seed maturation."

Similar revisions have been applied to other sections, especially in the subsections describing the TO-GCN networks. We now consistently separate the established biological roles of genes (supported by citations) from the novel observations provided by our study—such as their position in the TO-GCN hierarchy, their connectivity, and the predicted TF-binding sites. For instance, descriptions of senescence-associated or lipid-metabolism genes now explicitly note whether a mentioned function is based on literature or on our network/prediction results (see Lines 588–595 and 607–612).

Comment: "*The Discussion is perhaps a little long and descriptive and at times and rather too speculative. Given that a substantial component of the discussion covers cone browning and lipidomic profiling it would be useful to capture previous work focused on this.*"

Response: We sincerely thanks for your valuable comments and suggestions. We have undertaken a significant revision to make the Discussion more concise, less descriptive, and better anchored in existing literature, particularly concerning cone browning and lipidomic profiling. The key improvements in the revised Discussion compared to the original are as follows:

We have substantially streamlined and consolidated the descriptive sections of the Discussion, and speculative statements have been either removed or replaced with references to established concepts and prior studies. For example, we have added a

direct comparison with work on *P. tabuliformis* (Lines 832–837), citing evidence that membrane-lipid remodelling plays a critical role in maintaining seed vigour. This external validation strengthens the interpretation of our lipid-remodelling results.

In addition, our analysis of the regulatory networks now more explicitly refers to functionally characterized genes and pathways in model systems such as Arabidopsis thaliana (e.g., *WRI1*, *SPHK*, *EKI*).

Comment: "*It might also be useful to note that there is considerable variety in cone and seed maturity across conifer species, particularly the link between colour and seed quality.*"

Response: We sincerely thanks for your constructive comments.

Accordingly, we have revised the relevant paragraph in the Discussion section to explicitly acknowledge the considerable variety among conifer species. The specific modifications are as follows:

Line 747-756: "Because the relationship between cone color change and seed maturity varies considerably across conifer species… In some species (e.g., European larch), the development of markedly red cones signals the optimal seed harvest time.33 In contrast, we report that in *P. massoniana*, a transition from green to brown (browning) is a more reliable visual indicator."

Line 832-837: "Noteworthy is that, ultrasonic treatment has been observed to induce a rejuvenation effect on aged *P. tabuliformis* seeds, an outcome attributed to the remodeling of specific membrane lipid classes, such as sphingolipids [10]. This suggests that … whether induced by an external stimulus or an internal developmental program, is a cornerstone of seed vigor in pines."

Line 837-841: "Furthermore, despite differences in the overall lipid accumulation trends between *P. massoniana* and *P. armandii* … This suggests that the regulation of membrane lipid composition constitutes a conserved biological process in the seed maturation of pine species."

Some suggestions for potential improvements:

Comment 1: "*There are several places were linking statements are needed for the content to make sense, e.g., in the abstract (ln. 11) – "Given the importance of lipid metabolism in maturation, integrate physiological, lipidomic, and transcriptomic analyses of Pinus massoniana cones and seeds to construct co-expression networks to*

elucidate maturation mechanisms." Could perhaps read "Given the importance of lipid metabolism in maturation, we have integrated physiological, lipidomic, and transcriptomic analyses of Pinus massoniana cones and seeds to construct co-expression networks to elucidate maturation mechanisms."

Response: We sincerely thank you for these helpful suggestions. We have revised the Abstract in accordance with your suggestion. In addition, the entire manuscript has been carefully reviewed and edited to improve logical flow and ensure clear transitions between sentences and paragraphs.

Comment 2: *"Likewise, units are often given without context e.g. the abstract (ln. 16) "seed Vigor peaked at 117.5" does not reveal much without units or a definition of seed vigour. It is perhaps better to leave out units until they appear in the main text with some form of explanation."*

Response: We sincerely thanks for your constructive comments. The seed vigor index (V_i) is a dimensionless composite indicator, without unit. To prevent confusion, we have revised the abstract to describe the trend qualitatively: "… while seed vigor peaked at cone cracking."

Moreover, we have added the calculation formula of V_i, along with other germination parameters in the Materials and Methods section (Lines 212–244).

Comment 3: "*There appears is some confusion regarding lipids and fats and how these are defined. In the introduction, starting on ln. 39 cone seeds are described as being "rich in unsaturated fatty acids …… their lipid content can exceed 15%. Later, on the same page, ln. 50 it is stated that "fat content in mature seeds can exceed 60%. The commentary around these two statements is a little repetitive as well as contradictory.*"

Response: We sincerely thank you for pointing it out. During revising, this section of the introduction has been reorganized and rewritten. The specific sentences and the repetitive commentary mentioned are no longer present in the current version of the manuscript. Thank you once again for your valuable comments.

Comment 4: "*In the materials and methods, is there a simpler way to express "belonged to the same asexual lineages" (ln. 87)? I am not sure what is being implied here? Are these four trees clones derived from a single parental lineage or is there some*

other meaning being suggested by lineages?"

Response: We sincerely thanks for your valuable comments. To express this more clearly, we have revised the text in the Materials and Methods section as follows:

Line 163-166: "Four ramets of the same clone, exhibiting similar growth status, were selected for sampling and used as independent biological replicates (n = 4)."

Comment 5: "*In the materials and methods (ln. 87) it would be useful to define the age of the cones at collection as there is quite a lot of variation in the ages that gymnosperm cones reach maturity.*"

Response: We sincerely thanks for your constructive suggestions. We have now added this information to the Materials and Methods to provide a clear timeline of the cone development process in our study.

Line 170-171: "The cones—the development of which began in March of the previous year—were approximately 20–22 months old when collected."

Comment 6: "*Given the importance of the seed indexes such as germination potential, germination index, and vigour index in the results section, it would be useful if an explanation of these were given in the materials and methods. The reference given, the difficult to obtain standard (ln.102) does not allow assessment of what these indexes show. This makes it difficult to interpret the comments regarding the relevance of changes that occur after key seed factors are optimal (ln 400)*"

Response: We sincerely thanks for your constructive suggestions. We have supplemented the detailed descriptions of the calculation formulas and measurement procedures for seed indexes and vigour index in Materials and methods.

Line 212-244: "Subsequently, germination rate (G_r), germination potential (G_p), germination index (G_i), and vigor index (V_i) were determined pursuant to the standards GB/T 2772-1999 standards according to the following formulas:

$$G_r(\%) = {}^{n}/_{N} \times 100\%$$

$$G_p(\%) = {}^{n_p}/_{N} \times 100\%$$

$$G_i = \sum({}^{G_t}/_{D_t})$$

$$V_i = \sum({}^{G_t}/_{D_t}) \times L$$

where N and n represent the total number of seeds and number of germinated seeds,

respectively; np represents the peak number of germinations observed on a single day; Gt is the number of germinated seeds on the nth day after the start of germination, and Dt is the corresponding number of germination days; and L represents the seedling growth potential, expressed as average embryonic root length."

In brief …"

Comment 7: "*The first-time abbreviations are used in the text it would be useful to share what they are e.g., PCoA (ln. 274) and TO-GCN (ln. 303). The full names are given in the figure legends but for ease of understanding it would be nice to make this clear the first time they are read. The use of PCoA confused me as principal component analysis is defined as (PCA) on ln. 225.*"

Response: We sincerely thanks for your valuable comments and suggestions. We have carefully revised the manuscript to ensure that all abbreviations, including PCoA and TO-GCN, are explicitly defined upon their first occurrence in the text.

Line 257-259: "To visualize the global differences in gene expression profiles across different maturation stages, Principal Co-ordinate Analysis (PCoA) was performed.

Line 299: "Time-ordered gene co-expression network (TO-GCN)"

In our study, PCoA is applied to the transcriptome data (TPM values of DEGs) using the Bray–Curtis dissimilarity metric, which is well-suited for capturing compositional differences and nonlinear patterns in high-dimensional gene-expression profiles. Meanwhile, Principal Component Analysis (PCA) is used for the lipidomic data (peak intensity matrix) based on Euclidean distance, as it is effective for summarizing continuous, approximately normally distributed metabolite abundance data. Both methods are clearly described in the revised Methods section (Lines 257–259 for PCoA; Lines 297–298 for PCA).

Comment 8: "*The gene on ln. 486 should read WRINKLED1 (WRI1) rather than WEINKLED1. There is established knowledge on the role of this gene that is wider than Arabidopsis. Alternative references such as To et al. (2012) and Tan et al. (2023) would better reflect this.*"

Response: We sincerely appreciate your valuable comments and suggestions. In the revised manuscript, we have corrected the gene to *WRINKLED1* (*WRI1*) (Line 913). In additional, we have also cited these two references in Line 914.

Reviewer #4

"Cone maturation is accompanied by a series of physiological changes. This manuscript aims to investigate the changes in lipid composition and gene expression in ovuliferous scales and seeds during cone maturation using transcriptomic and metabolomic analyses, and to reveal potential gene regulatory networks involved in ovuliferous scale cracking and seed maturation. However, the manuscript is written in a rather rough manner. There are issues with figure citations, descriptions of figure results, and the use of abbreviations for specialized terms. In addition, the manuscript needs to provide further evidence to support the potential regulatory relationships between transcription factors and key target genes, rather than inferring them solely from gene co-expression networks. Therefore, the manuscript requires substantial revision. The specific issues identified in the manuscript are as follows:"

Response: We are thankful for the comprehensive and encouraging evaluation of our work. We would also like to acknowledge your work and dedication invested—it helped us to thoroughly improve the quality of our manuscript. We have addressed the specific points for improvement in detail below.

Comment 1: "*The abstract largely repeats the description of the results and lacks emphasis on the key points. In addition, the term 'glycerophospholipid' appears with two different spellings, as 'glycerophospholipid' and 'glycerol phospholipids."*

Response: We sincerely thanks for your valuable comments and suggestions. We have completely rewritten the Abstract to make it more concise and impactful, and we have standardized the terminology across the entire manuscript.

We have standardized the term for "glycerophospholipid" throughout the manuscript. All instances of "glycerol phospholipids" have been replaced with the correct and consistent spelling, "glycerophospholipids" (with the abbreviation GPLs introduced at its first occurrence).

Comment 2: "*Line 35-43: This paragraph does not align with the main theme of this article. Especially the last sentence: 'Despite this, pine seeds remain underexploited', I don't know what the author intends to convey.*"

Response: We sincerely thanks for your constructive comments. In the revised manuscript, we have removed this sentence and refocused the introductory paragraph to better align with the main theme of the study. The specific modifications are as

follows:

Line 50-59: "Pine trees (*Pinus* spp.) represent a significant source of raw materials for various industrial applications, such as papermaking, construction, and resin extraction. Their seeds represent a valuable biological resource with considerable development potential, being rich in lipids and a variety of unsaturated fatty acids such as linoleic acid (LA) and oleic acid. Lipid accumulation within seeds is closely associated…"

Comment 3: "*Line 44-57: According to this section, lipid biosynthesis plays an important role in seed maturation of gymnosperms. However, the authors should critically discuss the limitations of previous studies. In addition, when citing earlier work, the authors are advised to include the genus or species names to ensure that readers clearly understand the research context.*"

Response: We sincerely thanks for your valuable comments and suggestions. We have added the discussion of the limitations of prior research, noting that most studies have focused primarily on seed developmental stages while largely overlooking metabolic changes in cones during maturation. In the subsequent paragraph, we emphasize the significance of cone maturation. For detailed modifications, please refer to lines 56–115 of the revised manuscript.

Additionally, we have explicitly included the genus and species names (e.g., *Pinus koraiensis*, *Pinus tabuliformis* in lines 56–59; *Pinus mugo*, *Pinus nigra*, and *Pinus sylvestris* in lines 92–96) when referring to previous studies, which provides clearer context for readers.

Comment 4: "*Line 58-66: In this paragraph, no studies are cited that demonstrate a role of lipid biosynthesis and metabolism in cone maturation and dehiscence. The authors should cite relevant studies, if available.*"

Response: We sincerely thanks for your constructive comments. In the revised manuscript, we have incorporated relevant citations that document lipid metabolite composition and dynamics in cones of several pine species (e.g., *P. mugo*, *P. nigra*, *P. sylvestris*) across developmental stages (Sahin et al., 2017; Hofmann et al., 2020). Furthermore, lipid biosynthesis and metabolism play an important role in the regulation of plant senescence, which is a characteristic feature of cone maturation (Thompson et al., 1998; Guo et al., 2021). Based on these observations, we hypothesize that lipid

metabolism similarly plays a key role in the maturation and cracking of conifer cones. For detailed modifications, please refer to lines 92–106 of the revised manuscript.

Comment 5: "*Line 67-79: The authors should provide a clear overview of the current status of research on cone and seed maturation mechanisms in Pinus massoniana. In addition, the authors should clarify the limitations of previous studies and explain how this study addresses questions that remain unresolved.*"

Response: We sincerely thanks for your valuable comments and suggestions. In the revised introduction, we have added a dedicated paragraph summarizing the current state of research on *P. massoniana* cone and seed maturation. We explicitly note that while previous studies have linked lipid accumulation to seed maturation and described cone nutrient dynamics, the molecular mechanisms and gene regulatory networks coordinating cone dehiscence and seed maturation remain unclear. And then, we clarify how our study through integrated physiological, lipidomic, and transcriptomic analyses, aims to fill these gaps by identifying key regulatory modules and lipid dynamics underlying these processes. For detailed modifications, please refer to lines 116–153 of the revised manuscript.

Comment 6: "*Line 89-91: It is unclear whether the term 'scales' mentioned here refers specifically to the ovuliferous scale. Moreover, although scales and seeds were used as experimental materials, the manuscript consistently uses the term 'cone' instead of 'scale' in the results section (e.g., lines 249 and 253). The authors should clarify the intended meaning of 'scales' and maintain consistent terminology throughout the manuscript.*"

Response: We sincerely thanks for your valuable comments and suggestions. In the context of our study, the "scales" indeed refer to the ovuliferous scales of the cone, which are the specific tissue dissected from the cone for analysis alongside the seeds. We have made the following revisions to the manuscript to ensure clarity and consistency:

In the Plant materials section (Lines 176-179), we have explicitly defined the term at its first appearance: " Ovuliferous scales (hereinafter referred to as "scales") were carefully separated from the cone axis, immediately frozen in liquid nitrogen, and stored at –80°C for later transcriptomic and metabolomic sequencing."

We focused our analysis on ovuliferous scales because their morphological and

physiological changes—particularly the browning and eventual opening—serve as direct visual and functional markers of final cone maturation. Therefore, when discussing organ-level processes in the Results and Discussion, we use the term "cone" to maintain a coherent narrative of cone development as a functional unit. This terminology is consistent with several previous studies of cone physiology and maturation (Horstmann et al., 2022; Dawson et al., 1997; Madrigal et al., 2021), in which "cone" is used to represent the whole organ even when data derive primarily from scale tissues.

Comment 7: "*Line 137-140: This manuscript uses the genome of Pinus tabuliformis as the reference for mapping the transcriptome data of Pinus massoniana. The authors should report the mapping rate to ensure the reliability and reproducibility of the analyses.*"

Response: We sincerely thanks for your valuable comments and suggestions. In the revised manuscript, we have included additional data pertaining to the mapping rate: "Approximately 70% of the reads could be unambiguously mapped to the *P. tabuliformis* reference coding sequence (Line 433-435)".

Additionally, the quantification was conducted using Kallisto, which relies on pseudoalignment strategy. This method has been demonstrated to be less sensitive to sequence divergence compared to traditional alignment algorithms, making it particularly suitable for cross-species or inter-subspecific transcriptome studies. In our study, achieving a mapping rate of ~70% is both reasonable and reliable (References: Amanda et al. Insights into Conifer Giga-Genomes. Plant Physiology, 166(4): 1724–1732, 2014). This rate aligns with expectations for cross-genus or cross-species mapping within the Pinaceae family and is sufficient to capture the core transcriptomic landscape of *P. massoniana*, thereby ensuring the reproducibility and validity of our subsequent analyses.

Comment 8: "*Line 159-168: In this section of the manuscript, only the method for predicting transcription factor binding sites is described, while the approach for predicting interactions between transcription factors and their target genes is not explained. How did the authors obtain the promoter sequences of the target genes in Pinus massoniana?*"

Response: We sincerely thanks for your valuable comments and suggestions. In this

study, we used a homology-based cloning approach. Briefly, ~2,500 bp upstream regions of candidate genes were retrieved from the closely related *Pinus tabuliformis* genome and used to design gene-specific primers. These primers were used to amplify the corresponding promoter fragments from *P. massoniana* genomic DNA by PCR; the products were cloned and verified by Sanger sequencing. The confirmed *P. massoniana* promoter sequences were then used for subsequent motif and TF-binding analyses. The detailed protocol is now described in the Methods (Lines 330–336). The successfully cloned and sequenced promoter fragment has been included as Supplementary **Figure S4**.

Furthermore, in the Materials and Methods section, we also supplemented the approach for predicting interactions between transcription factors and their target genes.

Lines 336–341: "Extracted promoter sequences of all genes were scanned using putative TFBMs to identify significant binding sites. A gene was predicted to be a potential target of a specific TF if its promoter contained ⩾ 1 significant binding site (based on the PWM relative score) for that TF's motif."

Comment 9: "*Line 170: qRT-PCR validation included only 12 randomly selected genes and did not emphasize key lipid metabolism regulators. Could the authors clarify the selection criteria and consider validating core functional genes to strengthen the conclusions.*"

Response: We sincerely thanks for your valuable comments and suggestions. The initial 12 genes were carefully selected as representative candidates because they exhibited high expression levels in both cone and seed tissues. They also showed significant differential expression across multiple developmental stages in transcriptome data. These made them suitable for a preliminary, broad technical validation of the RNA-seq dataset.

Moreover, in order to strengthen the validation of functionally important genes, we have added another 24 key genes identified as core regulators of lipid metabolism by our integrated analysis. Consequently, a total of 36 key genes (16 associated with cone maturation and 20 with seed maturation) were validated by qRT-PCR using RNA extracted separately from cone and seed tissues (**Figure 8** and **Figure S6**). We have also added the rationale for gene selection to the revised manuscript at line 720-721.

In addition, the 36 genes were selected on the basis of their functional relevance to

cone or seed development. Genes validated in cones (e.g., *LOX4*, *ALDH*, *ACS*) are implicated in lipid oxidation and senescence-related pathways associated with cone maturation, whereas genes validated in seeds (e.g., *LAG1*, *PDA*, *SPHK*) encode enzymes central to storage-lipid biosynthesis, membrane-lipid remodelling and sphingolipid metabolism—processes that are important for seed maturation and vigour.

Comment 10: "*Line 220:"3076 positive and 2088 negative ion metabolites", how were those results obtained? The authors do not clarify to which parameter the reported "positive" and "negative" correlation' with metabolite accumulation refer. In addition, ion is any atom or group of atoms that bears one or more positive or negative electrical charges, the metabolites listed in Table S2 are not ion metabolites. The authors should revise the terminology to ensure accuracy and consistency throughout the manuscript.*"

Response: We sincerely thanks for your valuable comments and suggestions. With verification, the terms "positive and negative ion metabolites" were inaccurate statement. To address this concern thoroughly, we have made the following revisions to the manuscript:

In the Materials and Methods section, we have added a sentence at the beginning of this section to clarify the rationale for using both ionization modes.

Line 277-279: "To achieve comprehensive coverage of lipids with diverse chemical properties, the LC-MS analysis was performed in both positive and negative electrospray ionization modes."

In the Results section, we have rephrased the problematic sentence for accuracy and clarity.

Line 469-472: the sentence has been changed to " Of 5164 lipid metabolites identified in cones and seeds during maturation, 3076 were detected in positive ion mode (and 2088 in negative ion mode)."

Comment 11: "*Line 225: Some abbreviations in the manuscript appear only once and are not used thereafter; in such cases, abbreviations are unnecessary (e.g., line 225). In addition, some terms are abbreviated more than once (e.g., line 254 and 235). The authors should thoroughly standardize the use of abbreviations to ensure accuracy and improve readability.*"

Response: We sincerely thanks for your valuable comments and suggestions. We have removed all abbreviations that were only used once, and ensured that each term is

abbreviated only upon its first appearance and used consistently thereafter. We believe these changes have significantly improved the clarity and readability of the manuscript.

Comment 12: "*Line 228: The term 'differentially expressed metabolites (DEMs)' is used inappropriately. The authors should replace it with 'differentially accumulated metabolites' or 'differential metabolites,' The authors should avoid using the verb 'express' with 'metabolite,' as this usage is inappropriate and may confuse readers.*"

Response: We sincerely thank you for pointing it out. We apology for these oversights. As suggested, we have carefully replaced all instances of "differentially expressed metabolites (DEMs)" with "differentially accumulated metabolites (DAMs)" throughout the manuscript (e.g., in the Results section, Figure legends, and the Materials and Methods) to ensure terminological precision and avoid any potential confusion for the readers.

Comment 13: "*Line 234: 'from T1–T5' should be revised to 'from T1 to T5'.*"

Response: We sincerely thanks for your valuable comments and suggestions. We have revised the text as suggested and also checked and corrected any similar instances throughout the manuscript.

Comment 14: "*Line 331: The figure citations are incorrect, and moreover, the legend of Figure 6 does not correspond to the figure.*"

Response: We sincerely thank you for pointing out these errors. We have corrected the in-text citation on line 640: the reference to **Figure 6B** has been changed to **Figure 6D** to ensure it aligns with the corresponding description in the text. Additionally, the mismatched labels between **Figure 6B** and **6C** have been swapped, and the figure legend has been updated accordingly.

Comment 15: "*Line 357-381: Figure 7 is not cited in sequential order.*"

Response: We sincerely thanks for your valuable comments. We have thoroughly reviewed the manuscript and systematically corrected the in-text citations of all figures to ensure they are now presented in right order.

Comment 16: "*Line 441-418: The discussion on the nutritional and medicinal roles of LA and ALA, and other compounds is relatively long and somewhat diverges from*

the core mechanisms addressed in this study. Could the authors better link these points to the core mechanism of seed and cone maturation in P. massoniana."

Response: We sincerely thanks for your constructive comments. We have removed the extended discussion regarding the nutritional and medicinal roles of LA and ALA for human health. Instead, the revised text now directly links the accumulation of these polyunsaturated fatty acids to the core physiological mechanisms of seed and cone maturation in *P. massoniana.*

Line 787-800: "Notably, the levels of specific polyunsaturated fatty acids (PUFAs), including LA and ALA, increased significantly in later stages. Beyond their acknowledged nutritional value, the accumulation of these PUFAs is critically linked to the core physiological processes of maturation. In seeds, LA and ALA are key components of storage lipids and membrane GPLs, contributing to energy reserve composition and membrane integrity, which are fundamental to the establishment of germination capacity and vigor. Conversely, in cones, the data suggest that the metabolism of these PUFAs may be channeled towards pathways associated with senescence, potentially through the production of oxylipins or other bioactive molecules that influence aging processes."

Comment 17: "*Line 334: In Figure 6B, the lipoxygenase 4 gene is not shown; instead, the ACS gene is displayed. Furthermore, aldehyde dehydrogenase and short-chain dehydrogenase/reductase are mentioned in the text without providing their abbreviations, yet their abbreviations appear directly in Figure 8.*"

Response: We sincerely thanks for your valuable comments. We have conducted a comprehensive revision of Figure 6B.

Specifically, the previous version contained excessive extraneous information, which overshadowed critical data such as the key Lipoxygenase 4 (*LOX4*) gene. In response, we have isolated the less pertinent information into **Table S14** and emphasized the core genes in **Figure 6B**. Furthermore, we have incorporated a discussion regarding the *ACS* gene and its relevant background within the results section to provide a more thorough and nuanced narrative.

Additional, abbreviations for aldehyde dehydrogenases (*ALDH*) and short-chain dehydrogenases/reductases (*SDRs*) have been introduced upon their initial mention in the main text. Such issues have been addressed and revised throughout the entire manuscript. Thank you again for helping us improve the precision and quality of our

work.

Comment 18: "*Line 427: What is the concept and main evidence supporting 'lipid metabolic reprogramming'? In addition, how does lipid metabolic reprogramming differ from the 'membrane lipid remodeling' mentioned later, and based on the results, how can reprogramming be distinguished from remodeling?*"

Response: We sincerely thanks for your constructive comments.

1) By "lipid metabolic reprogramming" we refer to a coordinated, stage-dependent, and tissue-specific reconfiguration of whole-cell lipid metabolism during maturation. This encompasses altered flux through multiple lipid pathways (synthesis, interconversion and degradation) that changes the relative abundance of major lipid classes (e.g., storage lipids versus structural/membrane lipids) at the organ level.

2) Evidences in our dataset supporting reprogramming are as following: (i) Lipidome-level shifts. Global lipid profiling shows directed changes in lipid-class composition across developmental stages — for example, progressive accumulation of triacylglycerols (TGs) concurrent with declines or rearrangements of glycerophospholipids (GPLs) in cones, and distinct stage-specific accumulation patterns of TGs/DGs and particular GPL species in seeds (see **Figures 3–5**). (ii) Pathway-level coordination. KEGG enrichment of stage-specific gene modules indicates coordinated regulation of multiple lipid metabolic pathways (e.g., glycerolipid and glycerophospholipid metabolism) during maturation (**Figure 2**). (iii) Transcriptional control. The time-ordered gene co-expression network (TO-GCN) identifies core TFs and modules (e.g., MYB- and AP2-family nodes) whose dynamics coincide with these lipidome changes, indicating transcriptional reprogramming underlying the metabolic shift (**Figures 5–7**).

3) How "remodeling" differs from "reprogramming". (i) Lipid metabolic reprogramming means broad, systemic reallocation of lipid metabolism across classes and pathways (storage vs structural lipids, synthesis vs degradation) that changes the overall lipid landscape of the tissue. (ii) Membrane lipid remodeling characters a specific mechanistic component of reprogramming focused on compositional and structural changes to membrane lipids (principal GPL species, sphingolipids), which directly alter membrane properties and signaling.

4) We distinguish them in our results as following: Reprogramming is inferred from large-scale, coordinated changes across many lipid classes together with concurrent

shifts in transcriptional modules (global lipidomic trajectories + network/KEGG evidence; **Figures 2–5**). In practice, we identify reprogramming by: (i) statistically significant, stage-dependent shifts in major lipid classes and numerous species across the lipidome, and (ii) corresponding rewiring of co-expressed metabolic gene modules.

Remodeling is identified by targeted changes in membrane-associated lipid species (specific GPL and sphingolipid profiles), enrichment of membrane-biosynthesis/remodelling pathways at particular stages, and correlations between these membrane-lipid changes and membrane-linked physiological traits (e.g., seed vigour). Thus, remodeling is recognized as the membrane-centric subset of changes within the broader reprogramming signal (see **Figure 7** for membrane-associated pathway enrichment and **Figures 3–5** for species-level lipid shifts).

Comment 19: "*Line 372: In Figure 7B, the DGD1 gene is not shown. In addition, the groups of genes described in lines 370–373 and lines 488–490 are inconsistent, and Figure 7B also does not display the CCT and LPCAT genes.*"

Response: We sincerely thank you for pointing out this inconsistency. The previous mention of *DGD1*, *CCT*, and *LPCAT* was retained from an earlier analytical framework and was inadvertently not removed after we adopted a more stringent and updated network analysis pipeline. In the final version of our analysis, these genes are not supported as core regulators in the late maturation stage.

Accordingly, we have removed the unsupported statements regarding *DGD1*, *CCT*, and *LPCAT* from the revised manuscript. The Discussion has been carefully revised to focus exclusively on the core genes robustly identified by the final network analysis and presented in **Figure 7**, namely *PDA*, *LAG1*, *EKI*, and *SPHK*.

All relevant descriptions have been corrected consistently throughout the manuscript, as detailed below.

Line 682-686: "In late-maturation stages, … sphingosine kinase (*SPHK*), sphingosine N-acyltransferase (*LAG1*), phospholipid diacylglycerol acyltransferase (*PDA*), and ethanolamine kinase (*EKI*) were identified."

Line 919-921: "In the late stages of seed maturation within the co-expression network, the core lipid metabolism genes (e.g., *SPHK*, *EKI*, *PDA*, and *LAG1*) have been identified to ..."

Line 966-969: "We propose a model in which…*LAG1, PDA, EKI, SPHK*), associated with the accumulation of ..."

Comment 20: "*Line 480-482 and 500-502: Co-expression networks do not necessarily represent regulatory relationships. Although the methods section states that the JASPAR database was used to predict regulatory interactions between transcription factors and target genes, the key regulatory relationships mentioned in this section were not predicted. Additionally, during cone maturation, the regulatory interactions between key transcription factors (MYB/MORF4L/TRF/ERF) and key genes (ALDH/SDR/ACS) were also not predicted. The authors are advised to include these analyses.*"

Response: We sincerely thanks for your constructive comments and suggestions. We have conducted the TFBS prediction analysis in accordance with the recommendations, and the key results have been explicitly presented in the revised manuscript to substantiate the proposed regulatory relationships.

For the cone maturation network, the predicted regulatory interactions between the key senescence-associated TFs (e.g., *MYB, TRF, ERF*) and the key lipid oxidation-related genes (including *ALDH, SDR*, and *ACS*) are now summarized in **Figure 6E**. This new panel provides a clear schematic of the predicted TF-target gene relationships.

Similarly, the key regulatory relationships within the seed maturation module, including those involving TFs like *MYB*, *ARF*, and *ERF* their putative target genes (such as *GDP, PDA, LAG1*, etc.), are now illustrated in **Figure 7C**.

We have revised the text in the respective Results sections to directly guide the reader to these new figures:

Line 623-626: "Similarly, we inferred interactions between TFs and core lipid metabolism genes. The results suggest … (**Figure 6E**)."

Line 698-701: "Predicted interactions among these factors further support … (**Figure 7C**)."

Furthermore, to provide direct experimental support for our predicted regulatory relationships, we selected two representative transcription factor–target gene pairs — one derived from cone maturation process and one from seed — for functional validation by dual-luciferase reporter assays. Both the qualitative luciferase imaging and the quantitative reporter measurements confirmed that the tested TFs activate expression of their predicted target genes. These results are presented in **Figures 6F–H** and **7D–F** of the revised manuscript.

Comment 21: "*Line 529: Based on the results of this study, when is the optimal harvest timing for seeds in Pinus massoniana?*"

Response: We sincerely thanks for your constructive comments. Based on the results presented in **Figure 1**, the optimal harvest time for *P. massoniana* seeds is the cone cracking stage. Although seed germination capacity stabilizes after cone browning, the seed vigor index continues to increase and peaks during cone cracking (**Figure 1D**). We have clearly stated this conclusion in the conclusion section of revised manuscript.

Line 953-955: "We determine that the optimal time to harvest *P. massoniana* seeds is at the cone cracking stage to acquire the most vigorous seeds."

Comment 22: "*Figure 8: Why are the MYB genes absent from the 'Aging signal activation' section in Figure 8? According to the results and discussion sections, MYB genes appear to play an important role in cone senescence.*"

Response: We sincerely thanks for your valuable comments. In the original version of **Figure 8**, their omission from the 'Aging signal activation' section was an oversight in the model's visualization. We have now rectified this by adding MYB to the cone senescence section of the updated **Figure 9** (corresponding to the original Figure 8).

Response to Reviewers' Comments

Manuscript Number: COMMSBIO-25-7147B

"Lipid metabolism-driven regulatory networks involved in cone senescence and seed maturation in pine (*Pinus massoniana*)"

Reviewer #3

"I previously reviewed this manuscript, which describes a detailed analysis of lipid dynamics and their regulation during seed maturation and cone cracking in Pinus massoniana. Through integration of physiological measurements, lipidomics, and transcriptomics, the authors identify substantial reprogramming of lipid metabolism and propose a model linking lipid metabolic shifts with scale senescence and seed maturation. This manuscript provides novel insights of interest to those with both theoretical and applied interest in gymnosperm reproduction and conifer breeding.

I find that the issues I raised in the initial review have been substantially addressed. The manuscript is improved in both readability and clarity. Additional references now provide links to previous work addressing similar scientific questions. The improved figures and added supplementary material give increased understanding of the complex data sets presented.

While the discussion has been refined and is less speculative, there remains a tendency to infer functional significance from correlative observations without supporting experimental evidence. Statements such as "These results reveal lipid classes to regulate seed quality" (paragraph beginning Ln. 375) and "seed maturation requires coordinated shifts in lipid classes: TGs/DGs boost germination and GPLs enhance vigor" (Ln. 631) draw mechanistic conclusions that the data do not provide direct evidence for. As these are correlative findings, the language should be moderated accordingly. Confirming roles for these lipid classes in regulating seed quality or modulating germination traits would require further experimental work beyond the scope of this manuscript."

Response: We are thankful for the comprehensive and encouraging evaluation of our

work. We would also like to acknowledge your work and dedication invested—it helped us to thoroughly improve the quality of our manuscript.

As suggested, we have carefully moderated the language in both the Results and Conclusion sections in the revised manuscript. Specifically:

Line 387-390: "These results indicate that lipid classes are associated with seed quality traits, with TGs and DGs showing correlations with germination-related traits and GPLs being more closely associated with seed vigor. "

Line 642-645: "Cone senescence is characterized by triglyceride accumulation and GPL degradation, while seed maturation is associated with coordinated shifts in lipid classes, with TGs/DGs being positively associated with germination traits and GPLs with vigor-related traits. "

Line 18-22: "Lipidomic profiling reveals dynamic lipid reprogramming: cone senescence was associated with triglyceride accumulation and glycerophospholipid degradation, whereas seed maturation involved shifts in lipid classes, with triglycerides/diglycerides and glycerophospholipids showing distinct associations with germination capacity and vigor, respectively. "

Line 26-29: "Conversely, core genes involved in lipid metabolism, hormone-response factors, and key transcription factors (e.g., *bZIP1*) formed a central module that was closely associated with lipid fluxes and may contribute to the regulation of seed germination capacity and vigor. "

A couple of minor suggestions:

Comment 1: *"In the title at Ln. 388, please use "time-ordered co-expression networks" in full before introducing the abbreviation "TO-GCN", as this is its first appearance in the Results section."*

Response: We sincerely thanks for your valuable suggestions. We have revised the title at Line 388 (now Line 399 in the revised manuscript) to spell out the term in full at its first appearance in the Results section, which now reads: " Time-ordered gene co-expression network（TO–GCN）related to lipid metabolism "

Additionally, we have thoroughly reviewed the entire Results section to verify that

all other abbreviations are properly defined upon their first mention.

Comment 2: *"The above paragraph – starting at Ln 388 suggests the network includes DAMs expressed at various stages. Is this metabolite accumulation or a measure of expression?"*

Response: We sincerely thanks for your important clarification. In the original text, the verb "expressed" was incorrectly applied to DAMs. We have carefully revised the manuscript to distinguish between gene expression and metabolite abundance throughout. Specifically:

Line 403-407: "Lower tiers encompass TFs and non-TFs expressed during early maturation stages, along with DAMs that accumulate during the same period; higher tiers comprise TFs and non-TFs expressed during late maturation, together with DAMs that accumulate at these later stages."

Line 436: In the description of GPL dynamics (Figure 6C), we have corrected "time-dependent expression decline" to "time-dependent decline in abundance" to accurately reflect that GPLs are metabolites, not gene products.

Comment 3: *"The Materials and Methods section states that ovuliferous scales were extracted from the cone for use in expression analysis and lipid studies, and the term scales is used consistently throughout this section. However, the Results section shifts to referring to cones without explanation. To avoid confusion, it would be helpful to include a brief clarifying statement that explicitly addresses this terminology change."*

Response: We sincerely thanks for your valuable suggestions. To avoid confusion regarding terminology, we have added a brief clarifying statement in the Materials and Methods section. The revised text now reads:

Line 98-101: "Given that scales constitute the major structural and physiological component of the cone[5,12,13], these scale samples were regarded as representative tissues for cone-level analyses." Accordingly, "cone" is used throughout the Results and Conclusion sections from this point onward."
