# Supplementary material for: Lipid metabolism-driven regulatory networks involved in cone senescence and seed maturation in pine (Pinus massoniana)
Source: Commun Biol. 2026 May 18;9:1050. doi: 10.1038/s42003-026-10267-z (PMC13444107; doi:10.1038/s42003-026-10267-z)
Supplement: Supplementary file 2 [file 42003_2026_10267_MOESM20_ESM.pdf]

nature portfolio

Corresponding author(s): Feng Wu

Last updated by author(s): Apr 21, 2026

Reporting Summary

Nature Portfolio wishes to improve the reproducibility of the work that we publish. This form provides structure for consistency and transparency in reporting. For further information on Nature Portfolio policies, see our Editorial Policies and the Editorial Policy Checklist.

Statistics

For all statistical analyses, confirm that the following items are present in the figure legend, table legend, main text, or Methods section.

n/a	Confirmed	
☐	☒	The exact sample size (*n*) for each experimental group/condition, given as a discrete number and unit of measurement
☐	☒	A statement on whether measurements were taken from distinct samples or whether the same sample was measured repeatedly
☐	☒	The statistical test(s) used AND whether they are one- or two-sided<br>*Only common tests should be described solely by name; describe more complex techniques in the Methods section.*
☒	☐	A description of all covariates tested
☐	☒	A description of any assumptions or corrections, such as tests of normality and adjustment for multiple comparisons
☐	☒	A full description of the statistical parameters including central tendency (e.g. means) or other basic estimates (e.g. regression coefficient) AND variation (e.g. standard deviation) or associated estimates of uncertainty (e.g. confidence intervals)
☐	☒	For null hypothesis testing, the test statistic (e.g. *F*, *t*, *r*) with confidence intervals, effect sizes, degrees of freedom and *P* value noted<br>*Give P values as exact values whenever suitable.*
☒	☐	For Bayesian analysis, information on the choice of priors and Markov chain Monte Carlo settings
☒	☐	For hierarchical and complex designs, identification of the appropriate level for tests and full reporting of outcomes
☐	☒	Estimates of effect sizes (e.g. Cohen's *d*, Pearson's *r*), indicating how they were calculated

Our web collection on statistics for biologists contains articles on many of the points above.

Software and code

Policy information about availability of computer code

Data collection	We employed Adobe Photoshop 2023 (https://github.com/adobe-photoshop) software to facilitate the collection of the Lab color model data.
Data analysis	We used custom software OmicShare bioinformatics analysis platform (https://www.omicshare.com/), as well as Cytoscape (https://github.com/cytoscape/cytoscape), JASPAR (https://jaspar.elixir.no/), SPSS (https://github.com/IBMPredictiveAnalytics).

For manuscripts utilizing custom algorithms or software that are central to the research but not yet described in published literature, software must be made available to editors and reviewers. We strongly encourage code deposition in a community repository (e.g. GitHub). See the Nature Portfolio guidelines for submitting code & software for further information.

Data

Policy information about availability of data

All manuscripts must include a data availability statement. This statement should provide the following information, where applicable:

- Accession codes, unique identifiers, or web links for publicly available datasets
- A description of any restrictions on data availability
- For clinical datasets or third party data, please ensure that the statement adheres to our policy

The raw sequencing data have been deposited in the Genome Sequence Archive (Genomics, Proteomics & Bioinformatics 2025) at the National Genomics Data Center and are publicly accessible under accession number GSA: CRA033822 at https://ngdc.cncb.ac.cn/gsa. The lipidomic data have been deposited in the OMIX database under accession number OMIX: OMIX013199 and are also accessible via the same National Genomics Data Center portal. The source data underlying the

graphs in the main figures and supplementary figures can be found in Supplementary Data 16. All other data supporting the findings of this study are available from the corresponding author upon reasonable request.

Research involving human participants, their data, or biological material

Policy information about studies with human participants or human data. See also policy information about sex, gender (identity/presentation), and sexual orientation and race, ethnicity and racism.

Reporting on sex and gender	Research that does not involve human participants, their data, or biological materials.
Reporting on race, ethnicity, or other socially relevant groupings	Research that does not involve human participants, their data, or biological materials.
Population characteristics	Research that does not involve human participants, their data, or biological materials.
Recruitment	Research that does not involve human participants, their data, or biological materials.
Ethics oversight	Research that does not involve human participants, their data, or biological materials.

Note that full information on the approval of the study protocol must also be provided in the manuscript.

Field-specific reporting

Please select the one below that is the best fit for your research. If you are not sure, read the appropriate sections before making your selection.

☒ Life sciences ☐ Behavioural & social sciences ☐ Ecological, evolutionary & environmental sciences

For a reference copy of the document with all sections, see nature.com/documents/nr-reporting-summary-flat.pdf

Life sciences study design

All studies must disclose on these points even when the disclosure is negative.

Sample size	Sample sizes were determined based on prior experience and established standards in the field. For each experiment, at least three biological replicates per group were used, which is sufficient to assess the variability and detect meaningful differences in the measured physiological parameters.
Data exclusions	In physiological experiments, data points that deviated by more than two standard deviations from the group mean were considered statistical outliers and were excluded from analysis. This exclusion criterion was pre-established. For each experimental group, a minimum of three biological replicates were retained.
Replication	At least three biological replicates were used for all statistical analyses in biological experiments. For quantitative real-time PCR (qRT-PCR), each biological replicate comprised at least two independent technical replicates. All attempts at replication were successful.
Randomization	There was no group allocation which needed to be randomized.
Blinding	There was no group allocation which would have required blinding.

Reporting for specific materials, systems and methods

We require information from authors about some types of materials, experimental systems and methods used in many studies. Here, indicate whether each material, system or method listed is relevant to your study. If you are not sure if a list item applies to your research, read the appropriate section before selecting a response.

Materials & experimental systems

n/a	Involved in the study	
☒	☐	Antibodies
☒	☐	Eukaryotic cell lines
☒	☐	Palaeontology and archaeology
☒	☐	Animals and other organisms
☒	☐	Clinical data
☒	☐	Dual use research of concern
☐	☒	Plants

Methods

n/a	Involved in the study	
☒	☐	ChIP-seq
☒	☐	Flow cytometry
☒	☐	MRI-based neuroimaging

Plants

Seed stocks

The pant materials were collected from early November to the end of December 2023 in the seed orchard of P. massoniana at the state-owned forest farm in Huangping County, China. Cones were collected from the same healthy branch on November 1st (T1), November 16th (T2), December 1st (T3), December 16th (T4) and December 31st (T5), with three cones collected at each time point. One cone was dissected to collect seeds and scales, which were then placed in liquid nitrogen and subsequently stored at -80°C for transcriptomic and metabolomic sequencing. The remaining two cones were brought back to the laboratory for phenotypic assessment and other analyses.

Novel plant genotypes

No novel plant genotypes were used in this study.

Authentication

This study does not involve the validation of novel plant genotypes.
