# Supplementary material for: Lipid metabolism-driven regulatory networks involved in cone senescence and seed maturation in pine (Pinus massoniana)
Source: Commun Biol. 2026 May 18;9:1050. doi: 10.1038/s42003-026-10267-z (PMC13444107; doi:10.1038/s42003-026-10267-z)
Supplement: Supplementary file 3 [file 42003_2026_10267_MOESM2_ESM.pdf]

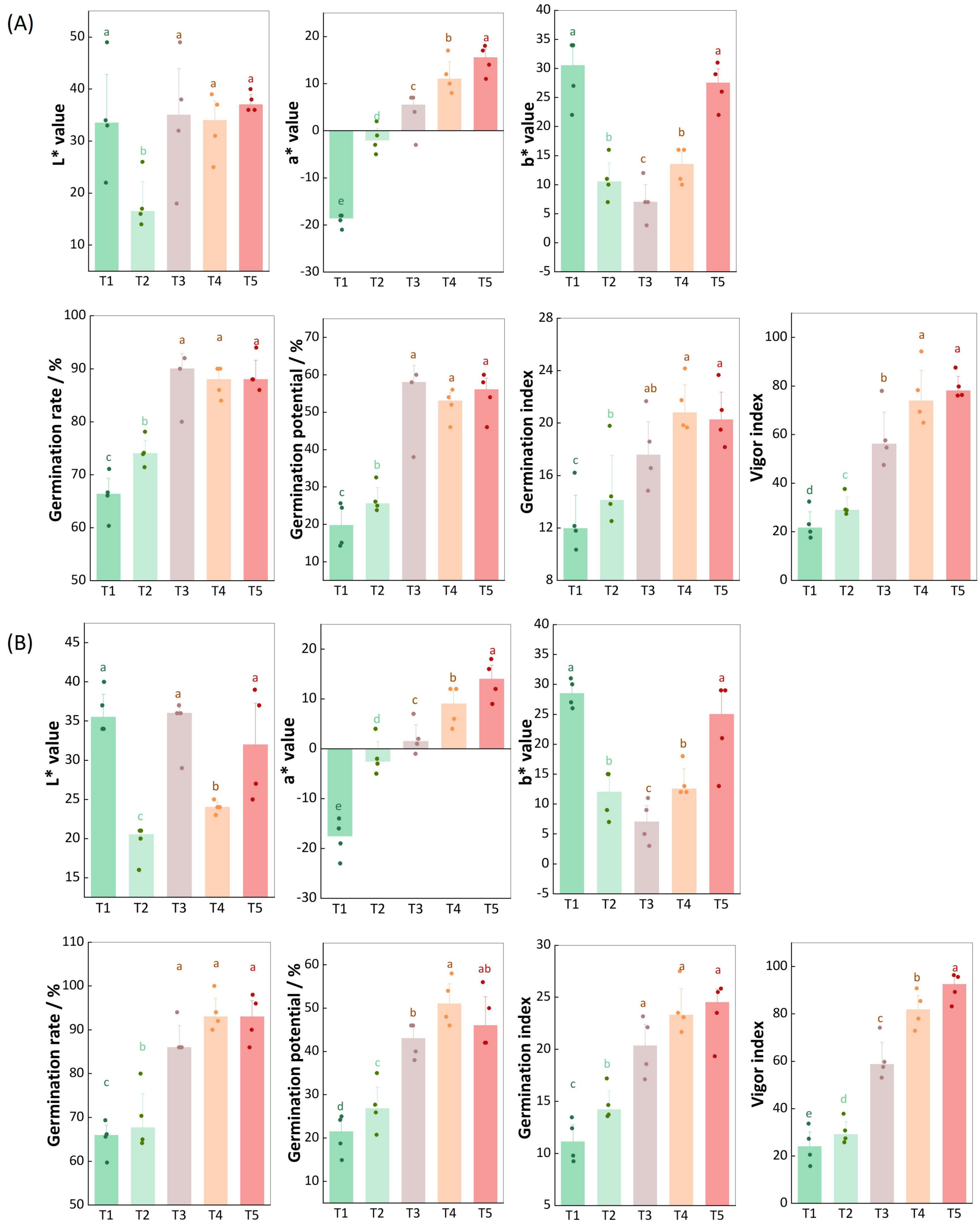

Supplementary Figure 1. Changes in cone color parameters and seed viability parameters at two additional *P. massoniana* seed orchards: (A) Huangping and (B) Duyun. Data are presented the mean ± SE (standard deviation) from four biological replicates (n = 4). Different letters denote statistically significant differences among stages (one-way ANOVA followed by Fisher's least-significant-difference [LSD] test; $p < 0.05$)

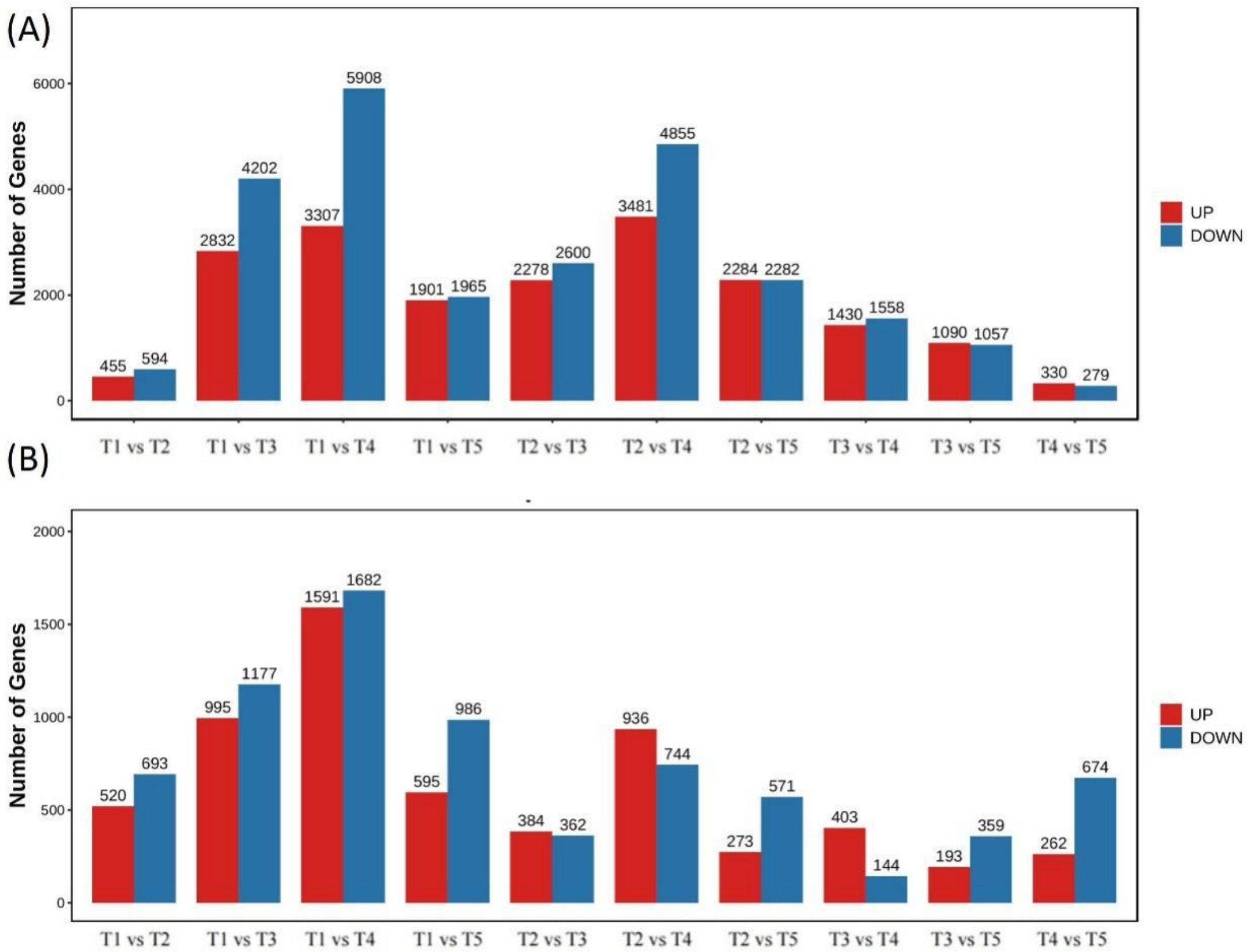

Supplementary Figure 2. DEGs of cone (A) and seed (B) of *P. massoniana* during maturation.

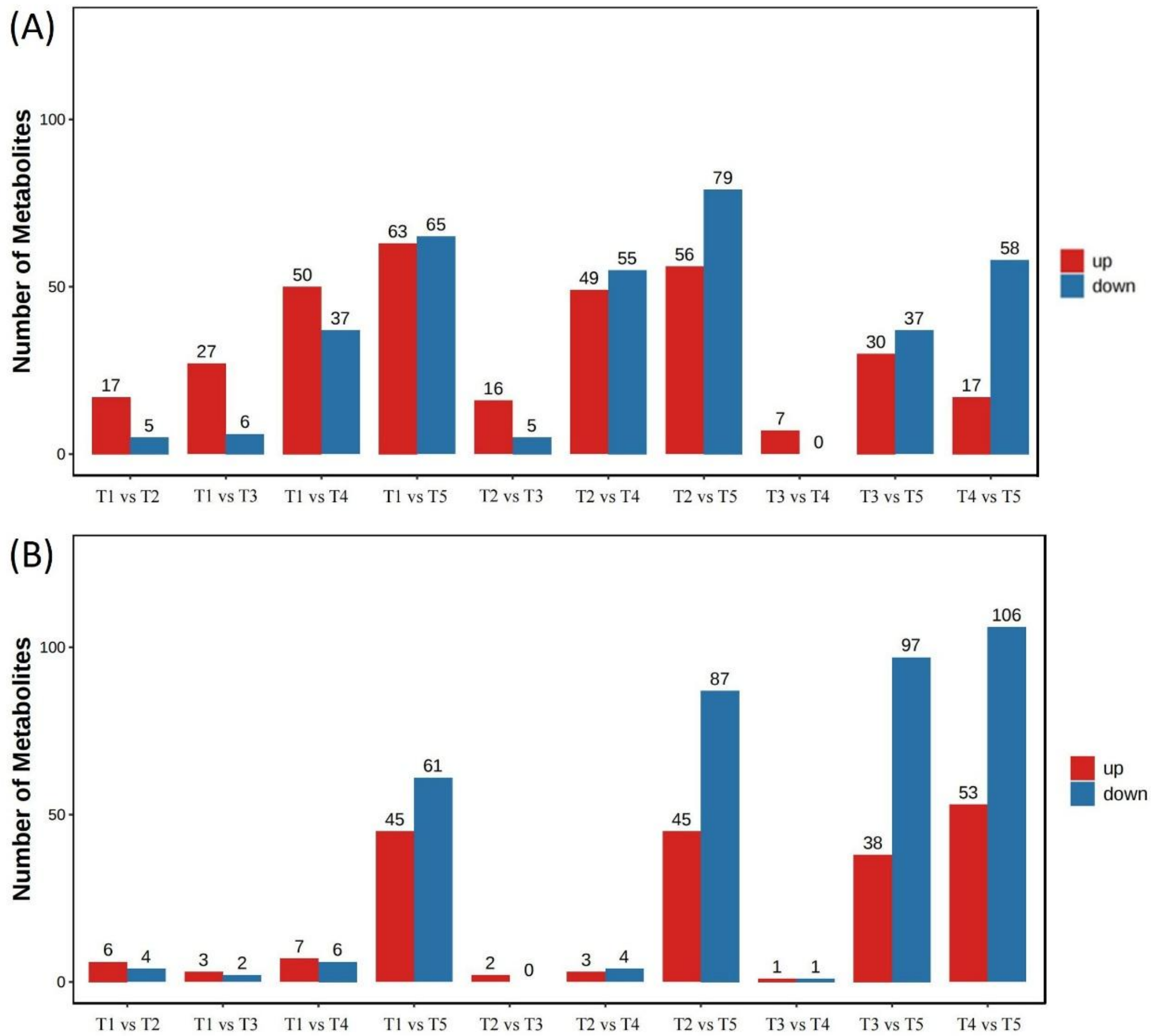

Supplementary Figure 3. DAMs of cone (A) and seed (B) of *P. massoniana* during maturation

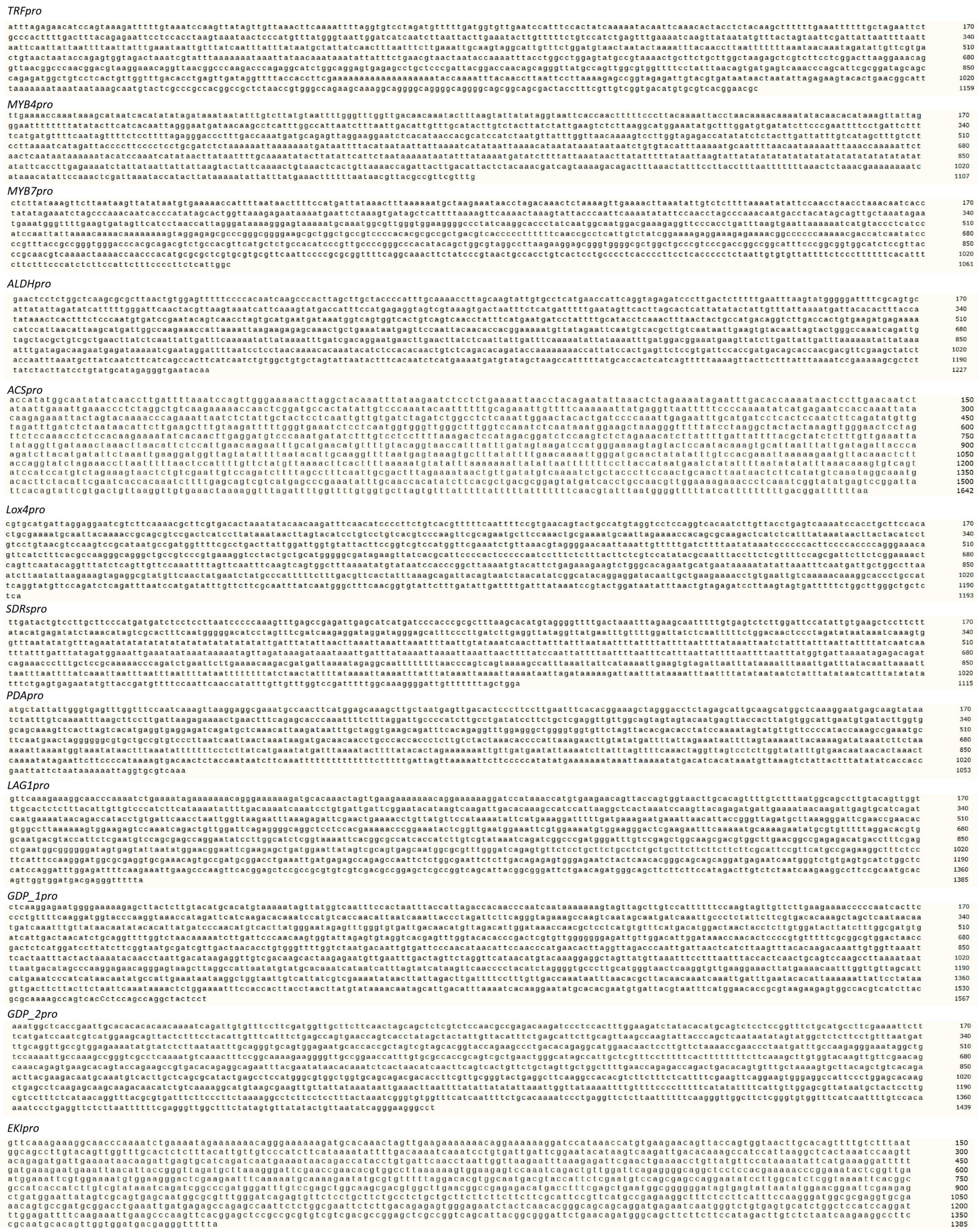

Supplementary Figure 4. Sequencing results of the promoter regions for twelve target genes.

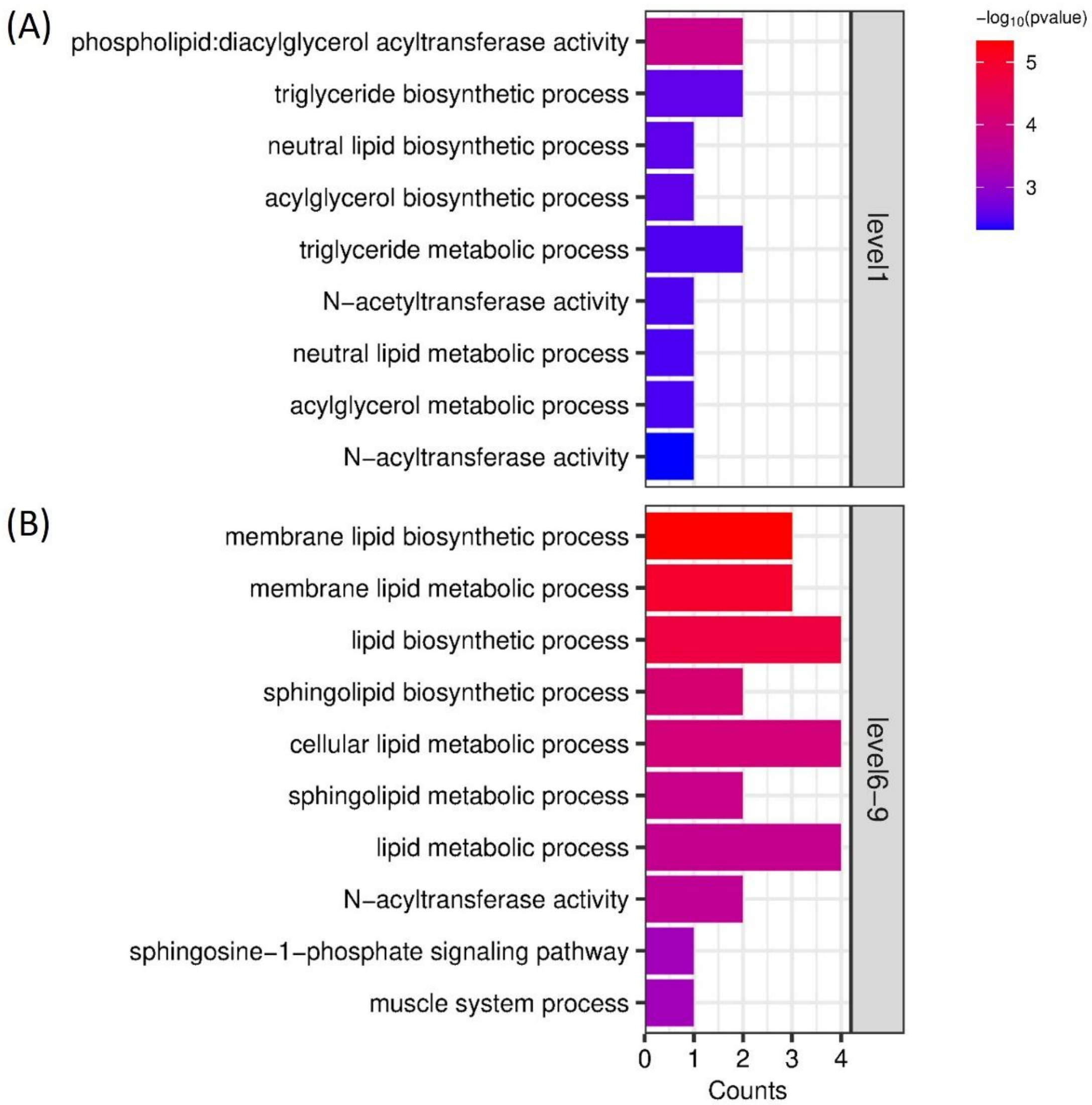

Supplementary Figure 5. GO enrichment analysis of genes related to the early (A) and late (B) stages of seed maturation.

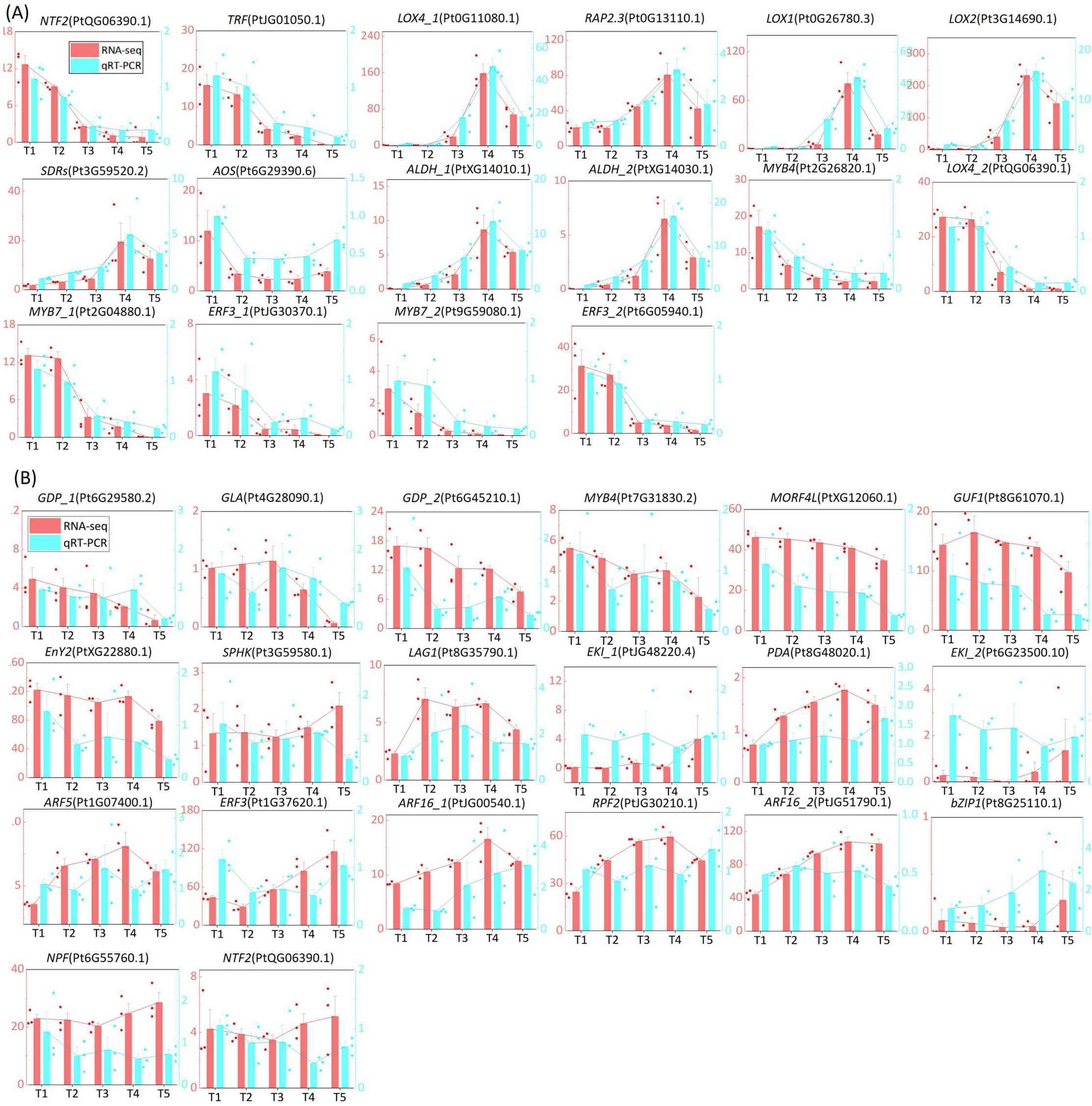

Supplementary Figure 6. qRT–PCR validation of key lipid metabolism-related DEGs. Key genes involved in the regulation of cone (A) and seed (B) maturation. The red and blue bars represent data from RNA-seq and qRT-PCR, respectively. The data represent the mean ± Standard error; biological replicates, n = 3.

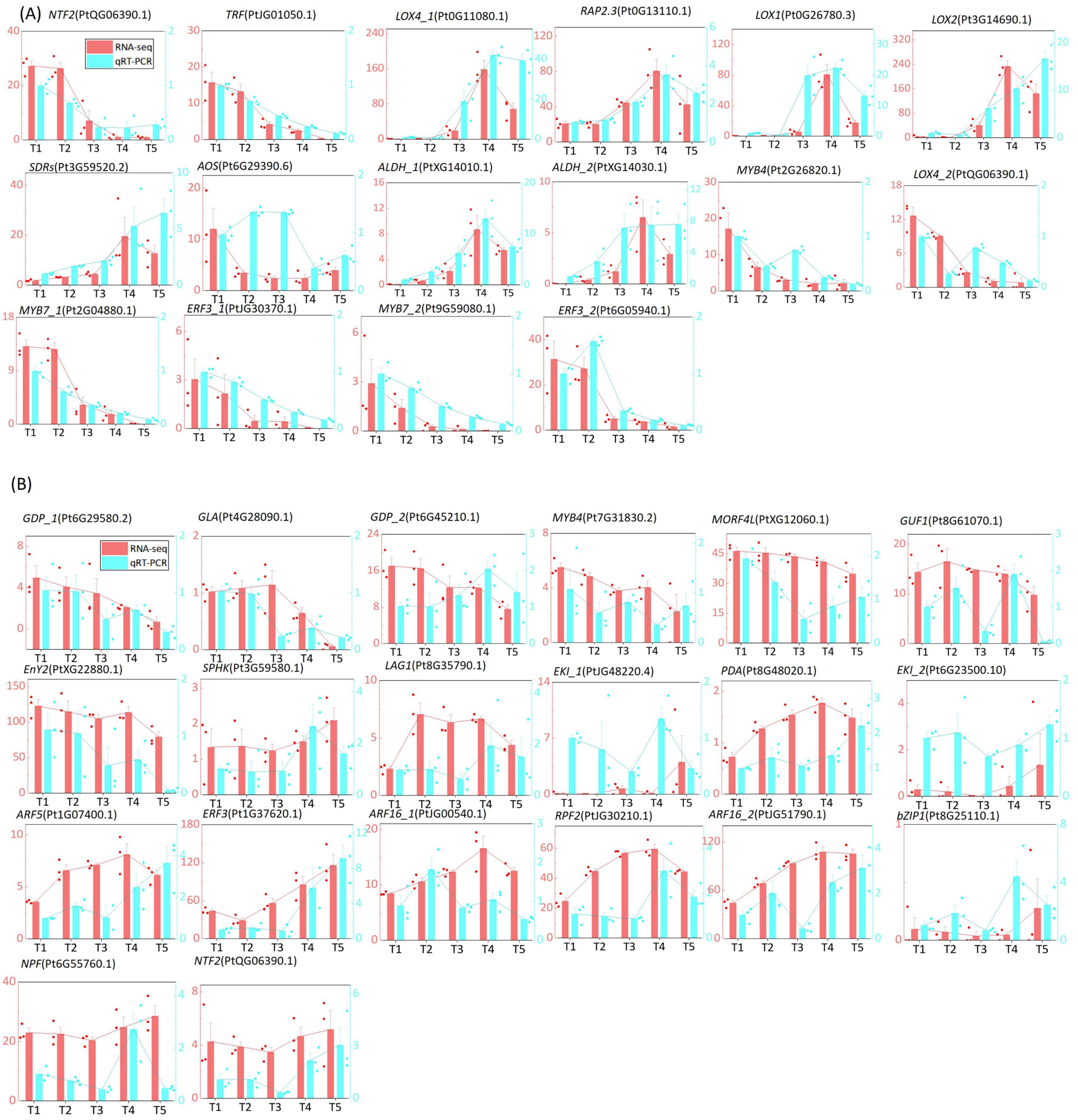

Supplementary Figure 7. qRT-PCR analysis was conducted to validate the expression levels of key genes using samples collected from additional sampling sites, with samples collected separately from the *P. massoniana* seed orchards in Duyun. Key genes involved in the regulation of cone (A) and seed (B) maturation. The red and blue bars represent data from RNA-seq and qRT-PCR, respectively. The data represent the mean ± Standard error; biological replicates, n = 3.
