# Supplementary material for: Lipid metabolism-driven regulatory networks involved in cone senescence and seed maturation in pine (Pinus massoniana)
Source: Commun Biol. 2026 May 18;9:1050. doi: 10.1038/s42003-026-10267-z (PMC13444107; doi:10.1038/s42003-026-10267-z)
Supplement: Supplementary file 4 [file 42003_2026_10267_MOESM3_ESM.pdf]

Description of Additional Supplementary File

File name: Supplementary data 1
Description: Meteorological data during the sampling period

File name: Supplementary data 2
Description: Summary of RNA-seq data filtering statistics

File name: Supplementary data 3
Description: Clustering of gene expression patterns and functional annotation of genes involved in cone maturation

File name: Supplementary data 4
Description: Clustering of gene expression patterns and functional annotation of genes involved in seed maturation

File name: Supplementary data 5
Description: KEGG pathway enrichment analysis of genes in modules with significant expression trends during cone maturation

File name: Supplementary data 6
Description: KEGG pathway enrichment analysis of genes in modules with significant expression trends during seed maturation

File name: Supplementary data 7
Description: KEGG Annotation of Lipid Metabolites

File name: Supplementary data 8
Description: Annotation information of conespecific TO-CCN-related factors

File name: Supplementary data 9
Description: Annotation information of seedspecific TO-CCN-related factors

File name: Supplementary data 10
Description: Correlation network of cone-specific TO-CCN-related factors

File name: Supplementary data 11
Description: Correlation network of seed-specific TO-CCN-related factors

File name: Supplementary data 12
Description: The types and quantities of TFs at each hierarchical level within TO-GCN

File name: Supplementary data 13
Description: The TO-GCN subnetwork regulating cone maturation

File name: Supplementary data 14
Description: The TO-GCN subnetwork regulating seed maturation

File name: Supplementary data 15
Description: Primer sequences for qRT-PCR validation and homology-based cloning of selected genes

File name: Supplementary data 16
Description: The source data behind the graphs in the paper
